# Micro‐Scale Topography Triggers Dynamic 3D Nuclear Deformations

**DOI:** 10.1002/advs.202410052

**Published:** 2025-01-28

**Authors:** Claire Leclech, Giulia Cardillo, Bettina Roellinger, Xingjian Zhang, Joni Frederick, Kamel Mamchaoui, Catherine Coirault, Abdul I. Barakat

**Affiliations:** ^1^ LadHyX CNRS Ecole Polytechnique Institut Polytechnique de Paris Palaiseau 91120 France; ^2^ Sorbonne Université INSERM UMRS‐974 Centre for Research in Myology GH Pitié‐Salpêtrière 47 bd de l'Hôpital Paris 75013 France

**Keywords:** endothelial cells, laminopathies, microgrooves, myoblasts, nuclear deformations, nuclear mechanics

## Abstract

Navigating complex extracellular environments requires extensive deformation of cells and their nuclei. Most in vitro systems used to study nuclear deformations impose whole‐cell confinement that mimics the physical crowding experienced by cells during 3D migration through tissues. Such systems, however, do not reproduce the types of nuclear deformations expected to occur in cells that line tissues such as endothelial or epithelial cells whose physical confinement stems principally from the topography of their underlying basement membrane. Here, it is shown that endothelial cells and myoblasts cultured on microgroove substrates that mimic the anisotropic topography of the basement membrane exhibit large‐scale 3D nuclear deformations, with partial to complete nuclear penetration into the microgrooves. These deformations do not lead to significant DNA damage and are dynamic with nuclei cyclically entering and exiting the microgrooves. Atomic force microscopy measurements show that these deformation cycles are accompanied by transient changes in perinuclear stiffness. Interestingly, nuclear penetration into the grooves is driven principally by cell‐substrate adhesion stresses, with a limited need for cytoskeleton‐associated forces. Finally, it is demonstrated that myoblasts from laminopathy patients exhibit abnormal nuclear deformations on microgrooves, raising the possibility of using microgroove substrates as a novel functional diagnostic platform for pathologies that involve abnormal nuclear mechanics.

## Graphical abstract

Microgroove substrates induce significant dynamic 3D nuclear deformations in different cell types. These deformations are principally driven by cell adhesion forces and are associated with rapid changes in perinuclear stiffness. The observation of abnormal nuclear deformations in cells derived from laminopathy patients opens the possibility of using microgroove substrates as a diagnostic platform for pathologies involving alterations in nuclear mechanics.

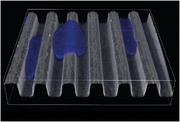

## 1 Introduction

Cells in vivo are often embedded in intricate environments whose organization imposes both physical guidance and mechanical confinement. Navigating these complex spaces requires extensive deformation of the nucleus, which is not only the largest organelle in the cell but also a major mechanostransducer.^[^ ^1^, ^2^, ^3^, ^4^ ^]^ The structural integrity of the nucleus relies principally on nuclear envelope proteins, most notably lamins, that provide a scaffold that safeguards chromatin organization, thereby ensuring genome integrity in the face of mechanical stress.^[^ ^5^, ^6^, ^7^ ^]^ It is therefore not surprising that altered nuclear mechanical properties are observed in several pathologies including laminopathies and cancer.^[^ ^8^ ^]^ In laminopathies, mutations in genes encoding A‐type lamins can lead to severe disorders such as cardiomyopathy, muscular dystrophy, or premature aging (progeria).^[^ ^9^, ^10^ ^]^ In the case of cancer, abnormal nuclear morphology is often a hallmark of the disease, and increased nuclear deformability correlates with heightened tumoral invasion.^[^ ^11^ ^]^

Different in vitro tools have been developed over the years to study nuclear deformations and rheology in various cell types.^[^ ^12^ ^]^ One class of such systems, which includes atomic force microscopy (AFM), optical or magnetic tweezers, and micropipette aspiration, enables the application of controlled forces that lead to local stretching or compression of individual nuclei, thereby providing precise mechanical characterization. These systems, however, suffer from several limitations including high experimental complexity, low throughput, and limited direct physiological relevance. Other systems, such as microfluidic channels with constrictions, constrain the cells uniformly over their entire contact surface and are typically designed to generate the types of nuclear deformations encountered by cells migrating through tight spaces, as would occur during immune or cancer cell intra‐ or extravasation. Missing from these systems is the ability to mimic the complex physical environment experienced by more quiescent, adherent cells such as epithelial or endothelial cells which, due to the topography and organization of the fibrous basement membrane on which they reside, are subjected in vivo to spatially non‐uniform subcellular contact stresses on their basal surfaces.

Systems that have targeted adherent cells to date have their limitations. For instance, wavy surfaces have been used to demonstrate that cellular deformations at the peaks and valleys can be transmitted to the nuclei, resulting in changes in nuclear morphology, chromatin organization, and gene expression;^[^ ^13^, ^14^ ^]^ however, the scale of the waviness in these systems leads to whole‐cell rather than subcellular contact stresses. Subcellular, nuclear deformations have been reported in cells cultured on micropillars;^[^ ^15^, ^16^, ^17^, ^18^ ^]^ however, these types of substrates fail to capture the anisotropy often present in the topography of basement membranes. In this context, we have been using microgroove substrates as idealized mimics of the anisotropic subcellular topography of the vascular basement membrane.^[^ ^19^ ^]^ Our recent studies using these substrates have demonstrated their ability to control vascular endothelial cell morphology, cytoskeletal organization, and collective migration.^[^ ^20^, ^21^ ^]^

In the present study, we explore the impact of microgroove topographic surfaces on cell nuclei. We show that microgrooves on the order of 5 µm in width, spacing, and depth elicit spontaneous, rapid, and robust nuclear deformations. Remarkably, these deformations include not only in‐plane nuclear elongation but also out‐of‐plane deformations that range from nuclei that partially penetrate the grooves to nuclei that are fully confined within the grooves. These 3D deformations are observed in a variety of cell types, and their extent can be controlled by modulating groove dimensions. We further show that these large‐scale 3D nuclear deformations are dynamic with nuclei going into and out of the grooves cyclically with no apparent DNA damage. Interestingly, AFM measurements demonstrate that these entry and exit cycles are accompanied by transient changes in perinuclear stiffness. Experimental observations corroborated by computational modeling demonstrate that the forces necessary for nuclear entry into the grooves are provided by cell membrane spreading and protrusion into the grooves, while cytoskeleton‐mediated forces surprisingly play a limited role. Finally, we show that myoblasts (muscle precursor cells) from patients with mutations in the *LMNA* gene cultured on the microgrooves exhibit abnormal nuclear deformations that can be detected and quantified using automated image analysis, thereby paving the path for the microgroove system to serve as a potential in vitro diagnostic platform for pathologies that involve abnormalities in nuclear mechanics.

## 2 Results

### 2.1 3D Nuclear Deformations on Microgrooves are Observed in Various Cell Types

**Figure 1:**
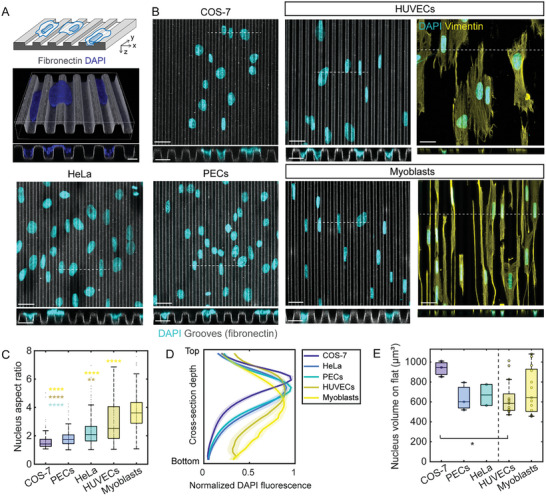
Nuclear deformations on microgrooves in different cell types. A) Schematic of cell culture on microgroove substrates (top). Confocal microscopy 3D reconstruction and cross‐section of HUVEC nuclei (blue) on microgrooves visualized using a fluorescent fibronectin coating (grey). Scale bar 5 µm (bottom). B) Z‐projection images (scale bar 25 µm) and cross‐sections (along dashed white lines; scale bar 10 µm) from confocal stacks showing nuclei (stained with DAPI, blue) after 8 h of culture on microgrooves (grey, width = spacing = depth = 5 µm, denoted as 5 × 5 × 5 µm) for COS‐7 cells, HeLa cells, parietal epithelial cells (PECs), human umbilical vein endothelial cells (HUVECs), and myoblasts. Right panels for HUVECs and myoblasts show immunostaining for vimentin to visualize cell shape. Scale bar 20 µm. C) Quantification of the nucleus aspect ratio on microgrooves for the different cell types (n = 109‐231 cells from 3 independent experiments). D) Quantification of normalized DAPI fluorescence intensity along the groove depth. N = 30 cells for each cell type. E) Nuclear volumes of cells on control flat surfaces (n = 2 to 13 independent experiments, 100–350 cells/condition). For all plots, one‐way ANOVA, Dunn's post‐test (^*^ *p* < 0.1; ^**^ *p* < 0.01; ^****^ *p* < 0.0001).

Microgroove substrates have been widely employed to generate cellular elongation and alignment, a phenomenon known as contact guidance.^[^ ^22^ ^]^ We have recently studied the mechanisms underlying contact guidance in human umbilical vein endothelial cells (HUVECs) cultured on fibronectin‐coated polydimethylsiloxane (PDMS) microgrooves (**Figure** **1**A, schematic).^[^ ^21^ ^]^ In the course of these investigations, we came across the fascinating observation that for a microgroove width and depth of ≈5 µm, the cell nuclei exhibited significant out‐of‐plane deformation and penetration into the grooves, in addition to the planar nuclear elongation and alignment associated with contact guidance (Figure 1A). Importantly, unlike studies on shallower grooves or planar adhesive lines where nuclear elongation is driven by overall cell elongation,^[^ ^23^, ^24^, ^25^ ^]^ the groove dimensions used here enabled complete confinement of the nucleus within the grooves (see Figure 1A) while the rest of the cell could remain outside the grooves. These large‐scale 3D nuclear deformations occurred very rapidly (as early as 30 min) after cell seeding and persisted for several days in culture, with the highest probability of occurrence between 2 and 24 h after cell plating. Additionally, nuclei of isolated cells exhibited greater deformations than those of cell monolayers (data not shown). These intriguing initial observations prompted us to further explore the phenomenon of nuclear penetration into microgrooves.

We began by testing if the observations initially made on HUVECs were unique to endothelial cells. To this end, we cultured different cell types on microgroove substrates and analyzed the behavior of their nuclei (Figure 1B). We observed different extents of nuclear elongation with nuclear aspect ratios ranging from 1.6 ± 0.6 in COS‐7 cells to 3.7 ± 1.2 in myoblasts (Figure 1C). Nuclear elongation was associated with at least partial nuclear penetration into the grooves in all cell types tested (Figure 1B, cross‐sectional views). To estimate the extent of nuclear penetration into the grooves, we quantified the average DAPI fluorescence intensity in different confocal z‐planes along the groove depth (Figure 1D). The analysis revealed that HUVECs and myoblasts, which showed the highest nuclear in‐plane elongation (Figure 1C), also exhibited fluorescence intensity profiles that were shifted further toward the bottom of the grooves than those of the other cell types tested (Figure 1D), indicative of deeper nuclear penetration into the grooves and hence more pronounced 3D deformations. Although this enhanced capacity for groove penetration was not correlated with nuclear volume (Figures 1B,E), the most pronounced nuclear penetrations into the microgrooves were typically associated with highly elongated cells, which was particularly evident in the case of myoblasts (Figure 1B, right panel). Because they exhibit the most pronounced 3D nuclear deformations, we opted to focus on HUVECs and myoblasts in all subsequent work.

### 2.2 Microgrooves Generate Different Categories of Nuclear Deformation that can be Modulated by Groove Dimensions

**Figure 2:**
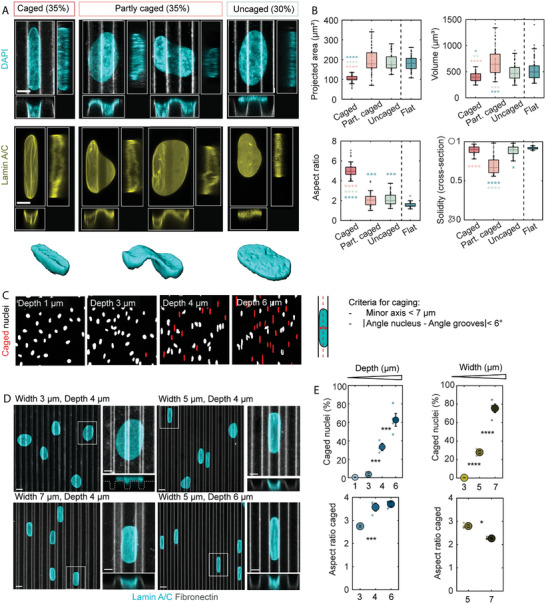
Characterization of nuclear deformations on microgrooves in HUVECs. A) Z‐projection images, cross‐sections, and 3D reconstructions of the different categories of nuclear deformation and their proportions observed on microgrooves (5 × 5 × 5 µm), with DAPI (top) or lamin A/C staining (bottom). Scale bar 5 µm. B) Morphological characterization of the different categories of nuclear deformation observed on microgrooves and control flat surfaces. Projected area and aspect ratio were quantified on z‐projection images, solidity (tortuosity) on the cross‐section images, and volume on 3D reconstructions; n = 42–50 cells/category from 3 independent experiments. C) Criteria for nuclear caging and output of the automatic detection of caged nuclei (red) for different groove depths (width = spacing = 5 µm). D) Nuclei (stained with lamin A/C, cyan) on microgrooves (grey) of different dimensions. Scale bars 10, 5 µm (zoom‐ins). E) Quantification of the percentage of caged nuclei and aspect ratio of caged nuclei for different groove depths (width = spacing = 5 µm) or groove widths (spacing = 5, depth = 4 µm). Dots represent individual experiments and error bars represent standard errors of the mean (SEM). n = 3–5 independent experiments. For all plots: one‐way ANOVA, Fisher's post‐test (^*^ *p* < 0.1; ^**^ *p* < 0.01; ^***^ *p* < 0.001, ^****^ *p* < 0.0001).

We have thus far seen that sufficiently deep microgrooves are capable of generating complex 3D nuclear deformations including both in‐plane elongation associated with contact guidance and out‐of‐plane nuclear penetration into the grooves. For simplicity, we will henceforth refer to the combination of both of these types of deformation as “nuclear deformations”. Careful observation of confocal images of nuclei on microgrooves revealed the coexistence of different categories of deformations (**Figure** **2**A) associated with specific morphometric features (Figure 2B). One category, termed “uncaged”, refers to nuclei that are elongated and oriented in the groove direction and that are suspended above the grooves. These nuclei remain flat with very little deformation in z, and they exhibit morphological features close to nuclei on flat control PDMS surfaces (Figures 2A,B). Note that this category was observed in HUVECs but not in myoblasts where all nuclei were at least partly deformed in z (Figure S1A, Supporting Information). On the other end of the deformation spectrum, nuclei can be fully confined within one groove (henceforth referred to as “caged” nuclei). These nuclei exhibit a very specific, highly elongated shape (aspect ratio of ≈5, Figure 2B) and are associated with a significant decrease in projected area and a loss of volume of ≈30% for HUVECs and ≈50% for myoblasts compared to nuclei on flat surfaces (Figure 2B; Figure S1B, Supporting Information). The remaining nuclei in the population, termed “partly caged”, exhibit an intermediate level of deformation and thus lie partly within either one or two grooves and partly on a ridge (Figure 2A). The partly caged nuclei are characterized by a highly tortuous contour as quantified by the solidity of the nuclear cross‐section (Figures 2A,B). Contrary to uncaged nuclei or nuclei on flat surfaces that are very flat and have smooth surfaces, caged and partly caged nuclei are thicker and exhibit surface wrinkles.

A notable advantage of the microgroove platform relative to other systems in which nuclear deformations have been studied is the generation of the deterministic, reproducible, and rather simple nuclear shapes described above. Consequently, the morphometric features identified here for the different categories of deformations allow us to directly infer the 3D deformation state from the 2D morphology of nuclei on standard wide‐field DAPI images without needing to resort to confocal microscopy. More specifically, caged nuclei can be automatically identified by applying a simple criterion on nuclear orientation and minor axis length (Figure 2C). The percentage of caged nuclei, which can thus be easily computed with high‐throughput, provides a simple and automatic quantification of the extent of nuclear deformation in microgrooves under different conditions. As already suggested in Figure 1D, quantification of caging frequency revealed higher caging percentages in myoblasts (≈70%) and HUVECs (≈35%) than in the other cell types tested (Figure S2, Supporting Information).

We next investigated the dependence of nuclear deformations on microgroove dimensions. For groove widths smaller than 4 µm, nuclei are unable to fully penetrate the grooves (except myoblast nuclei, possibly due to higher deformability, Figure S1C, Supporting Information). Increasing groove width from 5 to 7 µm increases the percentage of caged nuclei at the expense of partly caged and uncaged nuclei but also decreases their elongation (Figures 2D,E; Figures S1C,D, Supporting Information), suggesting that nuclei tightly conform to the groove space. For groove widths greater than 7 µm, the lateral confinement provided by the grooves is too mild to impose significant deformation of the cell nuclei (data not shown). Similarly, groove depths below 3 µm are too shallow to elicit considerable out‐of‐plane deformation and in‐groove penetration. Caging is first detected in 4 µm‐deep grooves and increases with groove depth, coupled with increased nuclear elongation until saturation for 6 µm‐deep grooves (Figures 2D,E; Figures S1C,D, Supporting Information). Thus, in HUVECs and myoblasts, the nuclear deformations described here occur over only a limited range of groove dimensions, namely 4–7 µm in both width and spacing and 4–6 µm in depth. Within this range, the type and extent of nuclear deformation can be controlled by choosing the appropriate set of groove dimensions for the considered cell type.

We then proceeded to examine the potential functional consequences of nuclear deformations on microgrooves. The EdU assay in HUVECs cultured on microgrooves of different depths revealed a slightly increased proliferation rate on microgrooves relative to cells on control flat surfaces (Figure S3A, Supporting Information). The observation of EdU‐positive nuclei and the witnessing of dividing caged nuclei during live‐cell recordings attest to the fact that nuclear deformations in microgrooves do not inhibit cell proliferation. We also examined potential DNA damage by immunostaining against phosphorylated H2AX (pH2AX), a commonly used marker for DNA repair after double‐strand breaks.^[^ ^26^ ^]^ Contrary to the positive control (etoposide‐treated cells), no nuclear pH2AX signal was visible in HUVECs on either flat substrates or microgrooves (Figure S3B, Supporting Information). In myoblasts, pH2AX‐positive cells were occasionally observed; however, this appeared to be equally frequent on flat substrates as on microgrooves, suggesting that the DNA damage is not related to the physical constraints on the nuclei imposed by the grooves. In both cell types, no occurrence of nuclear envelope rupture could be observed (data not shown). Thus, microgrooves constitute a tunable, high‐throughput platform that enables well‐controlled yet non‐detrimental nuclear deformations.

### 2.3 Nuclear Deformations on Microgrooves are Dynamic

**Figure 3:**
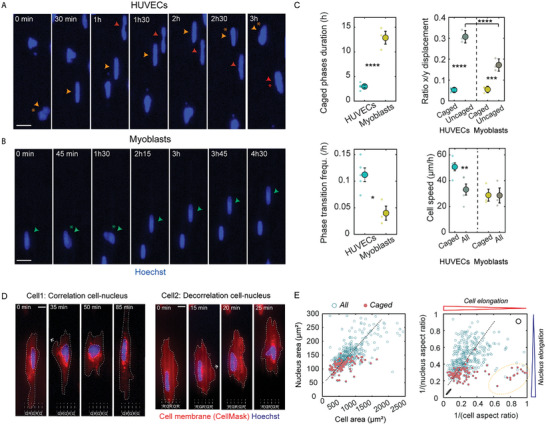
Dynamics of nuclear deformations on microgrooves. A,B) Images extracted from time‐lapse recordings of HUVEC or myoblast nuclei (stained with Hoechst, blue) on microgrooves (5 × 5 × 5 µm, oriented vertically). Arrowheads of the same color track the path of the same nucleus, and asterisks indicate uncaging phases. Scale bar 20 µm. C) Quantification of the x‐to‐y displacement ratio during caged or uncaged phases, mean duration of caging phases, frequency of transitions between phases (caging‐uncaging), and mean nucleus speed during caging phases compared to the mean speed of all nuclei at all times (“All”). Dots represent individual experiments, and error bars represent the standard error of the mean (SEM). n = 3 to 5 independent experiments. Student t‐test (2 groups) or one‐way ANOVA, Fisher's post‐test (^*^ *p* < 0.1; ^**^ *p* < 0.01; ^****^ *p* < 0.0001). D) Time‐lapse images of two representative cells (HUVECs) on microgrooves with the cell membrane in red (CellMask) and nucleus in blue (Hoechst). The white arrows show the extension of a cell protrusion. In both cases, initially caged nuclei become uncaged and move out of the grooves in the direction of the cell protrusion; this occurs more rapidly in Cell1 than in Cell2. Scale bar 10 µm. E) Cell area versus nuclear area and cell elongation versus nuclear elongation. Dots represent individual cells (n = 435 cells from 4 independent experiments), and caged nuclei are shown in magenta. Dashed black lines are for the eye only and do not represent fits of the data.

To further characterize nuclear deformations on microgrooves, we sought to determine the dynamics involved in nuclear caging. To this end, we used Hoechst to visualize nuclei in live cells and recorded their dynamics over a period of 24 h. Elongated, caged nuclei could easily be identified (**Figures** **3**A,B and Movies S1,S2, Supporting Information) and automatically detected in the recordings (see Experimental Section and Figure S4A, Supporting Information). Tracking individual nuclei revealed that caging is not permanent and that nuclei go in and out of the grooves cyclically. Each nuclear trajectory (see colored arrowheads, Figures 3A,B) can therefore contain caging phases separated by entry and exit phases (asterisks in Figures 3A,B) during which nuclei exhibit the characteristic morphology of the “partly caged” category. In HUVECs, caging phases are often separated by uncaged phases while in myoblasts, uncaged phases are virtually absent with nuclei rapidly deforming while moving from one groove to another between caged phases (Figure 3B). Quantification of the x‐to‐y displacement ratio for both the caged and uncaged phases shows that while nuclear movement during the caged phases is by definition predominantly along the groove direction (y‐direction) for both cell types, HUVECs explore more space perpendicular to the grooves (x‐direction) during the uncaged phases than do myoblasts (Figure 3C). In addition, the caging phases are significantly longer in myoblasts (≈13 h) than in HUVECs (≈3 h), and myoblasts correspondingly exhibit less frequent transitions among the different phases (Figure 3C). Interestingly, HUVEC migration speed is significantly higher during the caging phase than during the other phases; this effect is naturally not visible in the case of myoblasts whose nuclei are caged for the great majority of the recording time (Figure 3C). The observed differences in nuclear caging dynamics between HUVECs and myoblasts (short and cyclic in HUVECs vs long and stable for myoblasts) are consistent with the different proportions of caged nuclei observed in fixed samples for these two cell types (cf: Figure 2E; Figure S1D, Supporting Information).

To gain further insight into these nuclear dynamics, we subsequently examined the link between nuclear and overall cellular behavior. We therefore performed time‐lapse imaging of HUVECs on microgrooves with both nuclear (Hoechst) and cytoplasmic membrane (CellMask dye) staining (Figure 3D and Movies S3,S4, Supporting Information). These recordings revealed that nuclear movement overall follows cell movement, with nuclear exit from a groove preceded by the extension of a membrane protrusion into an adjacent groove (see arrows in Figure 3D). In most cases, a significant change in cell shape appears to be simultaneously (at least within the 5 min interval between frames) accompanied by a change in nuclear shape (see “Cell1” in Figure 3D and Movie S3, Supporting Information). However, in some cases, a time lag can be observed between cell and nuclear movement, with nuclei remaining caged for a period of time even after the cell has moved significantly in another direction (see “Cell2” in Figure 3D and Movie S4, Supporting Information). To better understand these observations, we analyzed the relationship between the morphological parameters of a cell and its nucleus on fixed‐cell images. Consistent with previous reports,^[^ ^27^ ^]^ we observed a positive correlation between cell and nuclear areas (Figure 3E, coefficient of correlation = 0.62) as well as cell and nuclear elongation (Figure 3E, coefficient of correlation = 0.3). When focusing specifically on caged nuclei, it can be seen that while most caged nuclei are found in elongated cells, some caged nuclei are nevertheless observed in more round cells (group within the dashed outline in Figure 3E), consistent with the two cases observed in the time‐lapse images.

### 2.4 Nuclear Deformations on Microgrooves are Associated with Changes in Perinuclear Stiffness

**Figure 4:**
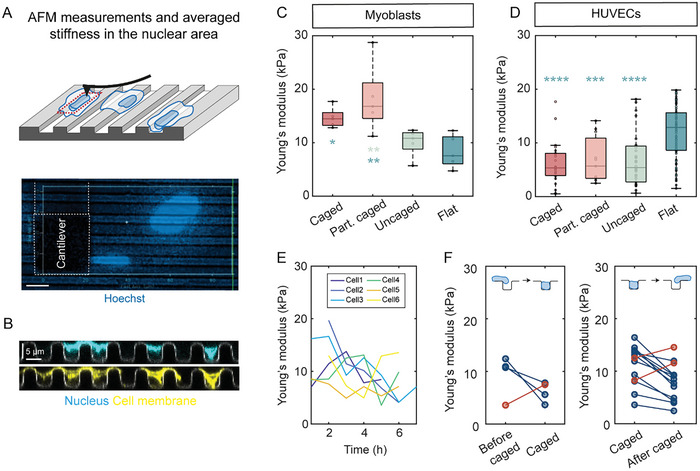
Nuclear deformations on microgrooves are associated with changes in nuclear stiffness. A) Top: Schematic of the atomic force microscopy (AFM) measurements; Bottom: image from the experiments showing a top view of the AFM cantilever and the nuclei stained with Hoechst. Scale bar 10 µm. B) Cross section from confocal microscopy images showing the localization of nuclei (cyan) and the cell membrane (yellow) C,D) Young's modulus values of nuclei in different categories of deformations on microgrooves or on flat PDMS surfaces, in myoblasts (left) and HUVECs (right). One‐way ANOVA, Fisher's post‐test (^*^ *p* < 0.1; ^**^ *p* < 0.01; ^***^ *p* < 0.001; ^****^ *p* < 0.0001); n = 3–6 independent experiments, 15–30 cells/category. E) Evolution of the Young's modulus of 6 different HUVEC nuclei with time. F) Evolution of Young's modulus of different HUVEC nuclei during transitions from uncaged to caged (left) or caged to uncaged (right). The red dots and lines correspond to nuclei that exhibit behavior different from that of the predominant trend 5–14 transitions from 3 independent experiments.

For a given applied stress, nuclear deformation (or strain) is determined by the mechanical properties of the nucleus. To probe the stiffness of nuclei during the dynamic nuclear deformations on microgrooves, we performed AFM experiments on both HUVECs and myoblasts with Hoechst‐stained nuclei to identify both the position of the nucleus and the category of nuclear deformation (**Figure** **4**A). In these experiments, measurements with a force setpoint providing an indentation of 0.5–1 µm were made every two pixels over the entire nuclear area. Because the nucleus is very closely apposed to the cell membrane in these very flat cells (Figure 4B), we believe that such indentations lead to significant probing of the nucleus. The Young's modulus was then extracted from the different force‐distance curves in accordance with the Hertz model (see Experimental Section for details) and averaged over the nuclear area. This parameter will henceforth be referred to as “perinuclear stiffness”.

We first made AFM measurements of nuclei on microgrooves in the different categories of deformation (caged, partly caged, or uncaged) as well as nuclei on flat surfaces. Myoblasts on flat surfaces were overall softer than on the microgrooves, while the opposite trend was seen for HUVECs (Figures 4C,D), which can be linked to different perinuclear structural reorganization upon culturing on microgrooves for the two cell types. In myoblasts, caged and partly caged nuclei were associated with significantly higher Young's moduli (14.6 ± 1.9 kPa and 18.2 ± 6.4 kPa, respectively) than uncaged nuclei or nuclei on flat surfaces (8.3 ± 3kPa) (Figure 4C). This result may reflect perinuclear structural remodeling and compaction as a result of caging (cf: Figure S1B, Supporting Information). In contrast, no significant stiffness differences among the three nuclear deformation categories were observed in HUVECs, principally due to the large spread in the measurements within each category (Figure 4D).

We hypothesized that the large variability in the measured Young's modulus within each nuclear deformation category in HUVECs may be linked to the highly dynamic cell and nuclear behavior in this cell type, which may have led to the possibility of including within one category nuclei that are in transition to another state. To test this idea, we made successive AFM measurements of single nuclei over time (every hour for 4 to 8 h) and found that perinuclear stiffness varies significantly in time within one cell (Figure 4E). Importantly, we were able to also monitor caging transitions during these measurements, with nuclei going into or out of the grooves. Interestingly, both of these transitions were associated with rapid changes in perinuclear stiffness, most commonly transient perinuclear softening (Figure 4F). These results suggest the existence of a cycle of changes in perinuclear stiffness associated with the cycle of nuclear deformations. Whether these changes in mechanical properties drive the observed nuclear deformations or are a consequence of these deformations remains to be determined.

We further considered two factors that might contribute to the measured stiffnesses. The first is a thickness effect whereby the Young's modulus in thin nuclear regions may be overestimated because the measurement may be polluted by the effect of the stiffer substrate and/or because of the limited validity of the Hertz model for thin substrates. However, for nuclei lying on both grooves and ridges, no visible differences in Young's modulus were observed between the regions above the ridges (1.6 µm‐thick on average) and those above the grooves (3–3.5 µm‐thick) (Figure S5A, Supporting Information). Therefore, we do not believe that the underlying substrate contributes significantly to the measured Young's moduli. The second factor that we considered is the possible contribution of perinuclear actin to the measurements. To test this notion, we treated the cells with low doses of the actin‐disrupting drugs latrunculin A and cytochalasin D (Figure S5B, Supporting Information). Perinuclear stiffness decreased with drug concentration, suggesting that perinuclear actin does indeed contribute to the measured perinuclear stiffness. It remains unknown at this point if this effect is direct due to the contribution of cortical actin above the nucleus to the measured stiffness or indirect, possibly due to the impact of actin disruption on the tension in the cell membrane and/or on the integrity and organization of other cytoskeletal networks in the cell. In any case, although actin disruption led to a shift in the measured Young's moduli, we nevertheless observed the same overall trends as those observed in untreated cells for the different categories of nuclear deformation, suggesting that the original measurements include a contribution of the nucleus intertwined with a contribution of perinuclear material.

### 2.5 The Cell Cytoskeleton is not the Principal Driver of Nuclear Caging in Microgrooves

**Figure 5:**
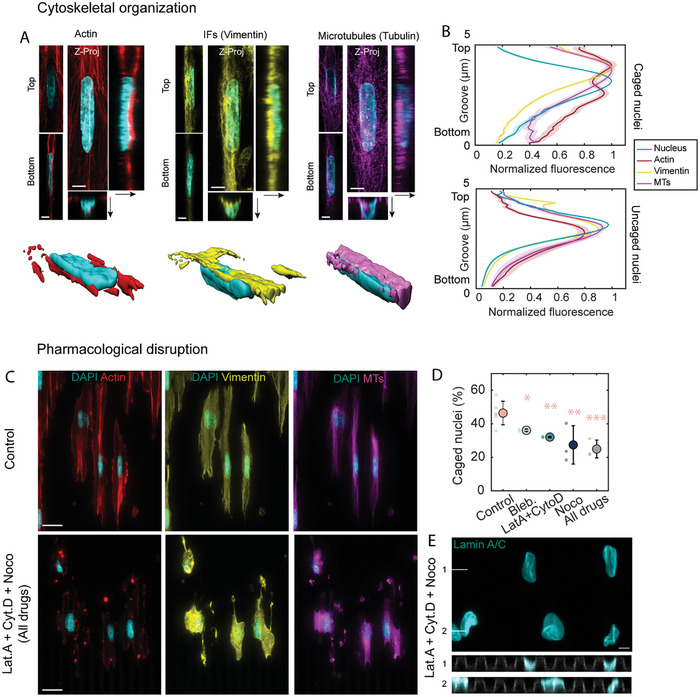
Organization and role of the cytoskeleton in nuclear deformations on microgrooves. A) Images from confocal stacks and 3D reconstructions showing the organization of actin (red), intermediate filaments (vimentin, yellow), and microtubules (α‐tubulin, magenta) around caged nuclei in HUVECs. Scale bars 5 µm. B) Quantification of the normalized fluorescence intensity for the three cytoskeletal networks and the nucleus as a function of groove depth for caged (top) and uncaged (bottom) nuclei. C) Immunostaining for actin (red), intermediate filaments (vimentin, yellow), and microtubules (α‐tubulin, magenta) in control cells or cells treated with latrunculin A (Lat.A), cytochalasin D (Cyto.D), or nocodazole (Noco) on microgrooves (5 × 5 × 5 µm, oriented vertically). Scale bar 20 µm. D) Quantification of the percentage of caged nuclei for different pharmacological treatments: control (DMSO), blebbistatin (Bleb.), latrunculin A and cytochalasin D (Lat.A + Cyto.D), nocodazole (Noco), or latrunculin A + cytochalasin D + nocodazole (All drugs). Dots represent individual experiments and error bars represent standard deviations. n = 3–6 independent experiments. One‐way ANOVA, Fisher's post‐test (^*^ *p* < 0.1; ^**^ *p* < 0.01; ^***^ *p* < 0.001). E) Z‐projection and cross‐sections of nuclei stained for lamin A/C and treated with latrunculin A + cytochalasin D + nocodazole. Scale bar 5 µm.

We next turned our attention to the mechanisms that underlie nuclear deformation on microgrooves. Because it links focal adhesions present at the bottom of the grooves to the nucleus, we hypothesized that the cell cytoskeleton may provide pulling forces that enable nuclear entry into the grooves. We first analyzed the 3D organization of the three main cytoskeletal networks: actin filaments, intermediate filaments (vimentin), and microtubules around caged nuclei in HUVECs (**Figure** **5**A). To this end, we quantified the localization of each cytoskeletal network relative to the nucleus by plotting its average fluorescence intensity as a function of groove depth (z) (Figure 5B). While some actin filaments were present at the ridge level, the most prominent actin stress fibers localized underneath caged nuclei, indenting the nuclear envelope in some cases. In contrast, a dense vimentin network was visible above the nucleus but was largely absent from the bottom of the grooves. Microtubules exhibited an intermediate localization, mainly present above the nucleus but also visible on the side and occasionally underneath caged nuclei (Figures 5A,B). This cytoskeletal organization was specific to caged nuclei, as illustrated by the different intensity profiles observed for caged and uncaged nuclei in Figure 5B. Similar results were found for myoblast caged nuclei (Figure S6A, Supporting Information).

To test the involvement of the different cytoskeletal networks in nuclear caging, HUVECs were incubated with different pharmacological agents: blebbistatin (100 µm) to decrease cell contractility, latrunculin A (10 nm) plus cytochalasin D (20 nm) to disrupt both cortical actin and actin stress fibers, or nocodazole (200 nm) to disrupt microtubules. The cytoskeleton‐disrupting agents were administered directly upon cell seeding to specifically establish their effect on nuclear entry into the grooves. Surprisingly, none of these treatments completely abolished nuclear penetration into the grooves. Microtubule disruption had the largest effect, but even then, a minimum of ≈30% of the nuclei remained caged (Figure 5D). To avoid a potential compensatory effect of any one of the cytoskeletal networks on the others and to target a minimalist system composed essentially only of the cell nucleus, membrane, and cytoplasm, we incubated the cells with a cocktail of latrunculin A, cytochalasin D, and nocodazole (20, 40, and 400 nm, respectively) (Figure 5C). While the effect of this treatment was drastic on the cells with complete loss of contact guidance (Figure 5C) and on nuclear morphology with more irregular shapes and wrinkling of the nuclear envelope, nuclear penetration into the grooves was not abolished (Figures 5D,E, cross‐sections). Similar results were obtained with myoblasts (Figure S6B, Supporting Information). In these cells, we further tested the involvement of the cytoskeleton by using myoblasts containing a mutation in the KASH domain of Nesprin‐1, a member of the Linker of Nucleoskeleton and Cytoskeleton (LINC) complex that links the actin cytoskeleton to the nuclear envelope (Figure S6C, Supporting Information). The mutation did not modify the occurrence of caging, suggesting that forces transmitted by the LINC complex are indeed not essential for nuclear caging.

### 2.6 Nuclear Deformations on Microgrooves are Driven by Cell Membrane Protrusion and Surface Adhesion Forces

**Figure 6:**
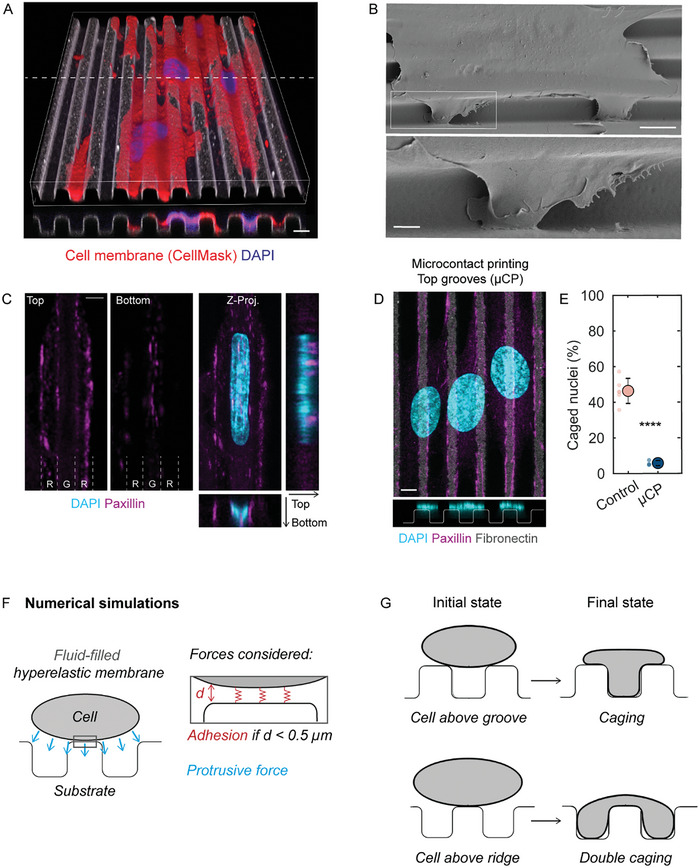
Influence of cell membrane behavior on nuclear deformations on microgrooves. A) 3D reconstruction and cross‐section (along the dashed white line) of HUVEC cell membranes (red, stained with CellMask) and nuclei (blue, stained with Hoechst) on microgrooves (5 × 5 × 5 µm). Scale bar 5 µm. B) Electron microscopy of a cell on microgrooves showing protrusions into the grooves. Scale bars, 10, 2 µm (inset). C) Immunostaining for paxillin (magenta) shows the presence of focal adhesions on both the ridge (R) and groove (G) surfaces. Scale bar 5 µm. D) Immunostaining for paxillin (magenta), DAPI (blue), and fibronectin (grey) shows nuclear behavior on microgrooves when adhesion is restricted to the top of the ridges (microcontact printing). Scale bar 5 µm. E) Quantification of the percentage of caged nuclei for a homogeneous fibronectin coating or microcontact printing experiments (µCP). Dots represent individual experiments and error bars represent standard deviations. n = 3–6 independent experiments. One‐way ANOVA, Fisher's post‐test (^****^ *p* < 0.0001). F) Schematic of the cell representation and forces considered in the computational model. G) Numerical simulations of cell deformations on microgrooves for different initial cell sizes and positioning.

The results described in the previous section indicate that cytoskeleton‐mediated forces are not essential for initiating nuclear caging. We therefore turned our attention to the potential role of cell spreading and membrane protrusions. In 3D reconstruction of either confocal or electron microscopy images, we observed large protrusions of the cell membrane along the groove walls down to the bottom of the grooves (**Figures** **6**A,B). Staining for focal adhesions (paxillin) confirmed that cells establish adhesions both on the ridge surfaces and at the bottom of the grooves, below caged nuclei (Figure 6C). To test if cell membrane protrusion and the formation of focal adhesions within the grooves were required for nuclear deformations, we used microcontact printing to restrict the fibronectin coating to the ridges while the remaining groove surfaces remained uncoated (and thus non‐adhesive) (Figure 6D). In this case, nuclear caging was essentially abolished (Figure 6E), indicating that cell membrane protrusion into the grooves and subsequent formation of focal adhesions on the groove surface is necessary for nuclear entry into the grooves. Because the formation of focal adhesions on the bottom surfaces of the grooves plays an essential role in nuclear caging, we wished to determine if the nuclear deformations reported here were specific to fibronectin‐coated microgrooves. To this end, we also tested microgrooves that were functionalized with either laminin or collagen IV. The results demonstrated nuclear caging in those cases as well (Figure S7, Supporting Information), suggesting that a wide range of integrins can be involved in this process.

To understand if cell spreading and membrane protrusions alone can indeed provide the driving force for cell and nuclear entry into the grooves, we performed computational simulations in which the cell was modeled simply as a fluid‐filled hyperelastic membrane with substrate adhesion and a downward‐acting force to represent membrane protrusion (see Experimental Section and Supporting Information for more details) (Figure 6F). The simulation results demonstrated that this minimalist model is sufficient to drive cell entry into the grooves. Depending on the initial cell size and positioning (centered on a groove or a ridge), both full caging and “double” caging can occur (Figure 6G) as seen in the experiments (cf: Figures 1, 2).

A limitation of the current computational simulations is the absence of a nucleus in the modeled cell. However, we would expect that as long as the intrinsic mechanical properties of nuclei allow them to deform sufficiently to fully penetrate the grooves, they would exhibit similar caging behavior to that seen in the simulations. To test the notion that nuclei are sufficiently deformable to penetrate the microgrooves, we seeded isolated myoblast nuclei on microgrooves and observed their behavior after 3 to 6 h in culture (Figure S8A, Supporting Information). Although full caging was not observed during that time period, significant penetration of the nuclei into the grooves was visible, demonstrating that nuclei on their own can deform sufficiently to penetrate the grooves. To further reinforce this idea and because longer experiments were not feasible due to the rapid deterioration of isolated nuclei, we performed computational simulations of the isolated nuclei experiment. In the simulations, the nucleus was modeled as a fluid‐filled hyperelastic membrane whose deformation was driven either by gravity alone or by gravity combined with low‐level adhesion to the substrate (to simulate non‐specific adhesion). In these two cases and for a physiological range of nuclear Young's moduli,^[^ ^5^, ^28^ ^]^ the simulations reproduced the experimental observations and yielded partial to complete penetration of nuclei into the grooves (Figure S8B, Supporting Information).

Given the results above, we propose a model whereby the cell membrane protrusive and adhesive forces are sufficiently large to drive most of the cell cytoplasm into the grooves, with nuclear intrinsic mechanical properties allowing the nucleus to passively follow while potentially also being pushed into the groove by the apical cell membrane as the cell spreads. Although cytoskeleton‐mediated forces do not appear to play a central role in inducing initial nuclear deformations on microgrooves, it is certainly possible that the cytoskeleton subsequently actively reinforces this process and may be an important player in nuclear exit from the grooves during the cyclic deformation behavior.

### 2.7 Myoblasts with *LMNA* Mutations Exhibit Altered Nuclear Deformations on Microgrooves

**Figure 7:**
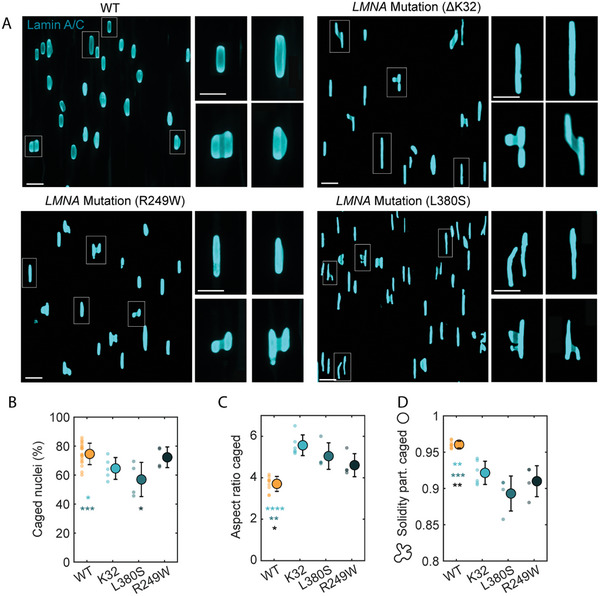
Myoblasts with *LMNA* mutations exhibit abnormal nuclear deformations on microgrooves. A) Nuclei from WT myoblasts or myoblasts carrying mutations in the *LMNA* gene (ΔK32, R249 W or L380S) on microgrooves, immunostained for lamin A/C. Insets show zoom‐ins on representative nuclei. Scale bars 30 µm, 20 µm (insets). B) Quantification of the percentage of caged nuclei. C) Aspect ratio of caged nuclei. D) Solidity of partly caged nuclei. Dots represent individual experiments and error bars represent standard deviations. n = 4–9 independent experiments. One‐way ANOVA, Fisher's post‐test (^*^ *p* < 0.1; ^**^ *p* < 0.01; ^***^ *p* < 0.001; ^****^ *p* < 0.001).

Various pathologies such as laminopathies and certain types of cancer are associated with alterations in nuclear mechanical properties.^[^ ^8^ ^]^ In light of the observed changes in nuclear stiffness that accompany nuclear deformations on microgrooves, we hypothesized that the microgroove platform may be useful in unveiling abnormalities in nuclear mechanical properties. To test this hypothesis, we cultured normal myoblasts as well as myoblasts derived from patients with muscular laminopathies on microgrooves. More specifically, we studied 3 different heterozygous mutations in the *LMNA* gene (which codes for A‐type lamins) that are known to be responsible for severe congenital muscular dystrophies. All mutant cells exhibited drastically different nuclear deformations on microgrooves compared to wildtype (WT) cells (**Figure** **7**A). Overall, the incidence of nuclear caging was smaller in mutants which instead exhibited more partly caged nuclei that were also highly tortuous (Figures 7B,D). When caging occurred, the nuclei of mutant myoblasts were significantly more elongated than those of the WT cells (Figure 7C). Time‐lapse imaging showed that mutant cells tended to migrate faster than WT cells and to exhibit faster, shorter but more frequent caged phases (Figure S9, Supporting Information). Interestingly, the microgroove system allowed the detection of differences among mutations. For instance, the ΔK32 mutation was associated with very long caged nuclei, while cells with the L380S mutation exhibited very tortuous partly caged nuclei. Overall, nuclei from patient cells appear to be more deformable than those in WT cells and are seemingly unable to maintain their structural integrity in response to the physical constraints imposed by the microgrooves. These results are in line with previous reports showing that nuclei in lamin A/C‐knockout cells^[^ ^29^ ^]^ or in cells with *LMNA* mutations that lead to muscular dystrophy are more deformable than those in normal cells.^[^ ^30^ ^]^ Thus, because they mechanically challenge nuclei, microgrooves can reveal functional abnormalities that can be automatically detected and quantified, paving the way for potential diagnostic applications.

## 3 Discussion

In many tissues, the basal surfaces of adherent cells are subjected to biophysical cues due to the multi‐scale topography of the underlying basement membrane.^[^ ^19^ ^]^ In this study, we used microgroove substrates to investigate nuclear deformations that adherent cells might experience in vivo in response to substrate‐derived micrometric anisotropic topographical cues. Understanding nuclear deformations is important as these deformations can potentially impact critical cellular functions including intranuclear organization, transcriptomic activity, and activation of downstream signaling events.^[^ ^14^, ^31^ ^]^

The principal novel finding of the current work is that in addition to the previously reported nuclear elongation in the direction of the microgrooves associated with cellular contact guidance, microgrooves induce significant out‐of‐plane nuclear deformations, with partial to full “caging” of nuclei inside the grooves. These 3D nuclear deformations occur for a specific range of groove dimensions (width, spacing, and depth between 4 and 7 µm) that elicit physical confinement of the nucleus while the remainder of the cell can remain outside the grooves. Different categories of nuclear deformation can be generated using the microgroove system, and the relative proportion of the different categories can be controlled by varying the groove dimensions, rendering the system easily adaptable to different experimental settings and cell types. In contrast to micropillar substrates which induce complex and random nuclear deformations, the principal advantage of the microgroove geometry lies in its ability to generate deterministic and reproducible nuclear shapes such as caged nuclei.

In adherent cells, cytoskeletal forces are usually the primary drivers of nuclear deformation.^[^ ^25^, ^32^ ^]^ The specific organization of cytoskeletal elements around caged nuclei with actin stress fibers principally below the nuclei and intermediate filaments and microtubules above and at the level of the nuclei initially suggested that actin cables might serve to pull the nuclei into the grooves from below while intermediate filaments and microtubules may push down from above. Surprisingly, however, extensive pharmacological disruption of the actin and microtubule cytoskeleton failed to abolish nuclear entry into and caging within the grooves. It should be noted that although intermediate filaments were not targeted directly in the pharmacological disruption studies, vimentin staining revealed that intermediate filaments were nevertheless markedly disrupted by the relatively high doses of actin‐ and microtubule‐disrupting drugs used. We note also that intermediate filaments have been shown in other studies to protect and maintain nuclear shape rather than induce nuclear deformation.^[^ ^33^, ^34^ ^]^ In another albeit different experimental setting, it has been reported that upon microdissection from the cell body, nuclei retain their original shape, indicating that nuclear deformation can be decoupled from instantaneous cell shape‐dependent cytoskeletal forces.^[^ ^35^ ^]^ Taken together, the various observations described above have led us to conclude that nuclear caging in our system only weakly relies on cytoskeleton‐generated forces, in contrast to studies on micropillars where nuclear deformations are driven by actomyosin pulling forces.^[^ ^32^ ^]^

The observations above suggest that a minimalist cellular system composed only of a cytoplasm housed within an adherent and protrusive cell membrane can generate sufficiently large cellular deformations and penetration into the grooves. In line with this model, we showed experimentally that preventing cell adhesion to the sides and bottom of the grooves entirely abolishes nuclear caging. Numerical simulations confirmed that adhesion and protrusion forces alone are sufficient for driving penetration of a fluid‐filled hyperelastic membrane into the grooves. Additionally, we observed both experimentally and numerically that isolated nuclei can at least partially enter the grooves as well, suggesting that they can passively follow the cell cytoplasm into the grooves. While our results show that entry and caging of nuclei inside grooves are mainly adhesion‐driven, we did not specifically address the mechanisms governing nuclear exit from the grooves, which may be more dependent on cytoskeletal‐associated forces.

A particularly intriguing attribute of the nuclear confinement reported here is its dynamic nature whereby nuclei repeatedly and cyclically enter and exit the microgrooves. Such dynamics have not been reported in other experimental settings that induce nuclear deformations and thus appear to be another distinguishing feature of the microgroove system. Interestingly, the observed dynamics vary among cell types, with HUVECs exhibiting shorter but more frequent caging phases compared to myoblasts which remain caged for significantly longer periods of time (13 h vs 3 h on average). Consistent with this observation, in fixed‐cell samples, ≈70% of myoblast nuclei on 5 µm‐deep grooves are caged versus ≈35% for HUVECs. These differences in nuclear deformation dynamics may stem from intrinsic differences in nuclear mechanical properties between the two cell types, as suggested by our AFM measurements showing that myoblast nuclei on flat surfaces are softer (Young's modulus of ≈8 kPa) than those of HUVECs (≈12 kPa).

In myoblasts, prolonged nuclear caging appears to be accompanied by perinuclear stiffening. Although we did not observe a significant change in DAPI intensity in caged nuclei, this may reflect a level of nuclear compaction. In HUVECs, the dynamic caging and uncaging events are associated with transient mechanical changes that most commonly take the form of perinuclear softening. The structural basis for these changes remains unknown but may be linked to dynamic reorganization and modification of the lamin network in the nuclear envelope and/or reorganization of the cytoskeletal networks close to the nucleus. Whether this form of mechanical adaptation is a cause or a consequence of nuclear deformation remains to be established, i.e., while these nuclear stiffness changes might passively arise from structural modifications associated with nuclear deformations, they may also more actively enhance the deformability of nuclei to facilitate their entry into or exit from the grooves, akin to cancer cell nuclei softening during transendothelial migration.^[^ ^36^ ^]^

A question that remains unanswered at this point is the potential existence of subpopulations within each cell type that exhibit different caging capabilities, possibly due to distinct intracellular organization and/or mechanical properties. Our measurements of caging dynamics and perinuclear stiffness over time suggest that a single cell can adopt a broad range of mechanical and nuclear deformation states, pointing preferentially toward a homogeneous population. Experiments such as single‐cell transcriptomics would nevertheless be interesting to shed light on the potential existence of distinct subpopulations.

Despite the large‐scale nuclear deformations and loss of nuclear volume reported here, microgroove‐induced nuclear caging does not elicit visible DNA damage, at least in cells without nuclear envelope mutations. This may be attributable to the dynamic nature of nuclear caging which potentially shields the nucleus from prolonged mechanical stress. The mechanical adaptation of the nuclei (softening) upon entering or exiting the grooves may also help. It is also possible that the cell types tested here express relatively high levels of lamin, allowing the nuclear envelope to resist large deformations without rupturing. Overall, this result contrasts with other systems such as compression devices or microfluidic channels with constrictions which often generate nuclear envelope rupture and DNA damage.^[^ ^37^, ^38^ ^]^ In those systems, it is important to note that the mechanical stresses are actively imposed onto the nuclei whereas the deformations in the case of the microgrooves are “self‐imposed”.

The microgroove dimensions used in this study are comparable to those of the micro‐scale topography of many basement membranes in vivo.^[^ ^19^ ^]^ It would therefore be particularly interesting to determine if the nuclear deformations reported here are also observed in vivo. One potentially physiologically important feature highlighted in this study is the mechanical plasticity of cells and their nuclei during deformation, which may constitute a mechanism for cells to efficiently adapt to their environment and limit nuclear and DNA damage. In the case of *LMNA* mutations present in laminopathies, the abnormal nuclear deformations observed in this study likely reflect a lack of this structural and mechanical plasticity, resulting in an inability of nuclei to cope with the physical constraints imposed by the extracellular environment. These defects can be particularly detrimental in cells residing in tissues subjected to high mechanical stresses, such as muscle. This notion of perinuclear mechanical adaptation on short time scales and its underlying mechanisms certainly merit further investigation.

Our results also show that nuclear caging phases are associated with more directed migration and increased cell speed. If the same occurs in vivo, then this would suggest that micrometric anisotropic topographical cues can act as tracks that guide cell migration and that nuclear caging in these environments can potentially promote the efficient escape of “leader cells”. This process would be particularly relevant in the context of morphogenesis, angiogenesis, wound healing, and tumoral invasion. In the field of vascular medicine, the augmentation of directed endothelial cell migration can potentially be exploited for the improved cellular colonization of implantable devices such as stents, valves, and endovascular grafts.

In vivo, adherent cells are typically subjected to multiple biophysical cues simultaneously. For instance, the rigidity of the basement membrane is an important feature to consider in addition to its topography. In the case of the vascular endothelium, the cells are additionally subjected on their apical surfaces to fluid mechanical shear forces due to the flow of viscous blood and to stretch due to the transmural pressure difference. Myoblasts are also subjected to high strains due to muscle flexing and contraction. Exploring the impact of combined biophysical loading on nuclear deformation in different cell types certainly merits future studies.

An association between pathologies and cellular mechanics has long been proposed and is now increasingly supported by evidence linking changes in cellular or nuclear mechanics to disease development.^[^ ^8^, ^39^ ^]^ Mechanical biomarkers therefore constitute a promising approach for the detection of some of these pathologies, and efforts have been made to develop experimental systems exploiting these novel biomarkers,^[^ ^40^ ^]^ including various types of microfluidic devices based principally on deformability cytometry (constriction deformability or fluid shear deformability^[^ ^41^ ^]^). In this context, because of their simplicity, robustness, and high‐throughput potential, microgroove substrates can constitute a valuable tool to functionally test the deformability of cells and their nuclei. As a proof of concept of this idea, we were able to use the microgrooves to demonstrate significant differences in nuclear shapes between healthy myoblasts and myoblasts carrying three different mutations in the *LMNA* gene that cause severe muscular dystrophies (laminopathies). Thus, microgrooves constitute a promising tool to functionally probe nuclear integrity in laminopathies, where determining the pathogenicity of diverse mutations can be challenging. Further investigations targeting other pathologies, such as cancer, will test the applicability range of this system, potentially opening new avenues for diagnostic and therapeutic applications.

## 4 Conclusion

We have used microgrooves that mimic the micro‐scale anisotropic topography of tissue basement membranes to show that substrate topography induces significant nuclear deformations in several cell types. These deformations lead to nuclei that either partially or completely penetrate the grooves while the remainder of the cells can remain outside the groove. The nuclear deformations are cyclic with nuclei periodically entering and exiting the microgrooves, and these deformation cycles are accompanied by large and rapid changes in perinuclear stiffness. The nuclear deformations are driven principally by cell membrane adhesive and protrusive forces rather than by cytoskeleton‐generated forces. Finally, the demonstration that cells derived from patients suffering from laminopathy, a disease that involves abnormalities in nuclear mechanics, exhibit abnormal deformations on microgrooves raises the intriguing possibility of using microgroove substrates as a novel functional diagnostic platform for the detection of pathological alterations in nuclear structure and mechanics.

## 5 Experimental Section

### Fabrication of Microgroove Substrates

The original microstructured silicon wafer was fabricated using photolithography by direct exposure of a layer of SU8 photoresist (MicroChem, USA) with a µPG machine (Heidelberg Instruments). After exposure to trichloro(1H,1H,2H,2H‐perfluorooctyl)silane (Sigma) vapor for 1 h, the original wafer was used to create polydimethylsiloxane (PDMS Sylgard 184, Sigma–Aldrich, ratio 1:10) replicates. To create the final coverslip on which the cells were cultured, liquid PDMS was spin‐coated at 1500 rpm for 30 s on the PDMS mold. Before reticulation overnight at 70 °C, a glass coverslip was placed on top of the PDMS layer. After reticulation, the glass coverslip attached to the microstructured PDMS layer was gently demolded with a scalpel and isopropanol to facilitate detachment. Microstructured coverslips were then sonicated for 10 min in ethanol for cleaning and finally rinsed with water.

### Cell Culture

Microgroove substrates were incubated for 1 h with 50 µg mL^−1^ fibronectin solution (Sigma F1141) at room temperature after a 30 s plasma treatment. Mixing of fibronectin with fluorescent fibrinogen 647 (Thermofisher F35200) was used to visualize fibronectin localization. For other types of coating, the microgroove substrates were incubated for 45 min with 75 µg mL^−1^ laminin‐511 (Merck CS226326) or collagen IV (Southern Biotech 1250‐01S) at 37 °C. All cell types were cultured at 37 °C in a humidified atmosphere of 95% air and 5% CO_2_. Before culture, cells were detached with trypsin (Gibco, Thermo Fisher Scientific) and seeded onto microgroove coverslips at densities of 30000–50000 cells cm^−2^ for 2 to 24 h.

### Cell Culture—Endothelial Cells

Human umbilical vein endothelial cells (HUVECs, Lonza) in passages 4–8 were cultured in an EGM2‐MV medium (Lonza).

### Cell Culture—Myoblasts

Muscle biopsies were obtained from the Bank of Tissues for Research (Myobank, a partner in the EU network EuroBioBank, Paris, France) in accordance with European recommendations and French legislation. Following muscle biopsies, muscle cell precursors were immortalized as previously described.^[^ ^42^ ^]^ Control myoblasts were immortalized from one healthy subject. Immortalized human myoblasts carrying the following heterozygous mutations responsible for severe congenital disorders were also used: *LMNA* c.94_96delAAG, p.Lys32del (hereafter referred to as ΔK32), *LMNA* p.Arg249Trp (hereafter referred to as R249 W), *LMNA* p.Leu380Ser (hereafter referred to as L380S) and *SYNE‐1* homozygous c.23560 G<T, p.E7854X leading to a stop codon in exon 133 and deletion of the carboxy‐terminal KASH domain (hereafter referred to as Nespr‐1ΔKASH). Myoblasts were cultured in growth medium consisting of 1 vol 199 Medium/4 vol DMEM (Life Technologies, Carlsbad, CA, USA) supplemented with 20% fetal calf serum (Life technologies, Carlsbad, CA, USA), 5 ng mL^−1^ hEGF (Life Technologies, Carlsbad, CA, USA), 0.5 ng mL^−1^ βFGF, 0.1 mg mL^−1^ dexamethasone (Sigma–Aldrich, St. Louis, MO, USA), 50 µg mL^−1^ fetuin (Life Technologies, Carlsbad, CA, USA), 5 µg mL^−1^ insulin (Life Technologies, Carlsbad, CA, USA), and 50 mg mL^−1^ Gentamycin (Gibco, Life Technologies, Carlsbad, CA, USA).

### Cell Culture—Other Cell Types

Primary mouse Parietal Epithelial Cells (mPECs)^[^ ^43^ ^]^ (Material Trade Agreement from M. Moeller, University Hospital of the Aachen University of Technology) were isolated using transgenic mouse lines and fluorescence‐activated cell sorting (FACS). mPECs were cultured in supplemented ECBM (supplement kit Promocell) with Pen‐Strep and 20% FBS until 70% of confluence before passaging.

*HeLa and COS‐7 cells* were purchased from ATCC and cultured in DMEM‐glutamax with 10% FBS and Pen‐Strep.

### Cytoskeletal Disruption

The following cytoskeletal pharmacological agents were used: blebbistatin (Sigma B0560) at 100 µm, latrunculin A (Millipore 428026) at 10 nm (HUVECs) or 1 µm (myoblasts), cytochalasin D (Sigma C2618) at 20 nm, (HUVECs) or 1 µm (myoblasts) and nocodazole (Sigma M1404) at 0.2 µm. When used together, the following concentrations were used: latrunculin A 20 nm, cytochalasin D 40 nm, and nocodazole 0.4 µm. Drugs were incubated directly with the cells upon seeding and maintained for 90–120 min. Controls were treated with the equivalent DMSO concentration.

For AFM experiments, HUVECs were treated with 5 or 50 nm of latrunculin A + 10 or 100 nm of cytochalasin D (low and high doses, respectively). Myoblasts were treated with 50 or 100 nm of latrunculin A + 50 or 100 nm of cytochalasin D (low and high doses, respectively). Drugs were incubated with the cells cultured overnight and maintained during AFM measurements (2 h in general).

### EdU Assay for Cell Proliferation

Cell proliferation was assessed using the “Click‐iT Plus EdU imaging” kit (ThermoFisher C10640) after 24 h of culture, with an EdU incubation time of 7 h.

### Microcontact Printing

Flat PDMS blocks were used as stamps. After incubation with fibronectin (50 µg mL^−1^) mixed with fibrinogen 488 in PBS for 1 h at room temperature, stamps were rinsed once with water and dried with an air gun. Microgroove coverslips were plasma‐activated and were rapidly placed in contact with the stamps for 3 min under a 30 g mass, which deposited fibronectin only on the groove ridges. Cells were then cultured on the surfaces of the microgrooves as described above.

### Isolation of Cell Nuclei

All steps were carried out at 4 °C. After PBS wash, nuclei were isolated in a homogenization buffer containing 10 mm HEPES and 1 mm DTT. Cells were homogenized with ≈25 strokes using a Dounce homogenizer, collected in the homogenization buffer, and loaded over a 30% sucrose gradient. The samples were mixed thoroughly by inverting and then centrifuged at 800 g for 10 min, yielding a crude nuclear pellet. The pellets were resuspended in 10 mm HEPES/1 mm DTT, centrifuged again, and resuspended in HEPES/DTT. Nuclei were then plated on microgrooves and cultured for 3 to 6 h.

### Immunostaining

Culture coverslips were fixed with 4% paraformaldehyde (Thermo Fisher) in PBS for 15 min. After 1 h in a blocking solution containing 0.25% Triton and 2% bovine serum albumin (BSA), cultures were incubated for 1 h at room temperature with primary antibodies as follows: mouse anti‐laminA/C (Sigma SAB4200236), mouse anti‐ phospho‐histone H2AX (Merck 05–636), mouse anti‐paxillin (MA5‐13356, Thermofisher), mouse anti‐vimentin (ab8069, Abcam) or rabbit anti‐vimentin (ab92547, Abcam), mouse anti α‐tubulin (Sigma, T5168), All antibodies were diluted 1/400 – 1/200 in a solution containing 0.25% Triton and 1% BSA. Coverslips were washed three times with PBS and incubated for 1 h at room temperature with Alexa Fluor 555‐conjugated donkey anti‐rabbit antibody (ab150074, Abcam) or Alexa Fluor 488‐conjugated donkey anti‐mouse antibody (ab150105, Abcam) and DAPI. When needed, actin was stained during this last step using phalloidin (LifeTechnologies).

### Microscopy—Epifluorescence and Confocal Microscopy of Fixed Samples

Epifluorescence images were acquired on an inverted microscope (Nikon Eclipse Ti) with a 20X objective (Nikon Plan Fluor NA = 0.5). Confocal microscopy images were acquired on an inverted TCS SP8 confocal microscope (Leica) using a 63X objective. 3D reconstructions were performed using the IMARIS software.

### Microscopy—Scanning Electron Microscopy

Cultures were fixed in 2% glutaraldehyde in 0.1 m PBS at pH 7.4. They were dehydrated in a graded series of ethanol solutions and then dried by the CO_2_ critical‐point method using EM CPD300 (Leica Microsystems). Samples were mounted on an aluminum stub with a silver lacquer and sputter‐coated with a 5 nm platinum layer using EM ACE600 (Leica Microsystems). Acquisitions were performed using a GeminiSEM 500 (Zeiss).

### Microscopy—Live Cell Imaging

To stain the nuclei in live cells, Hoechst 33342 (Sigma H3570) diluted 1/5000 in PBS was incubated for 3 min with cells. For visualization of the cell membrane, Cell Mask Orange (ThermoFisher C10045) diluted 1/1000 in culture medium was incubated for 5–10 min with cells. Subsequent live recordings of HUVECs were performed using an automated inverted microscope (Nikon Eclipse Ti) equipped with temperature and CO_2_ regulation and controlled by the NIS software (Nikon). Images were acquired with a 20X objective (Nikon Plan Fluor NA = 0.5) for 3 h at 5 min intervals.

### Microscopy—Atomic Force Microscopy

For AFM measurements, nuclei were stained with Hoechst 3 h before the beginning of the measurements. Experiments were performed on a NanoWizard 4 BioAFM scanning force microscope (JPK/Bruker) mounted on a Nikon ECLIPSE Ti2‐U fluorescent microscope. A probe with a circular symmetric rounded tip with a typical radius of curvature of ≈30 nm (0.06–0.18 N m^−1^) (uniqprobe qp‐BIOAC‐CI, from NANOSENSORS) was used. The spring constant was determined upon calibration by the thermal noise method. Quantitative imaging (QI) (JPK, Berlin, Germany) was conducted in water at 37 °C, with a setpoint of 0.8 nN for 90 ms (corresponding to an indentation of ≈0.5–1 µm). Young's modulus values were extracted using the Hertz model (Poisson's ratio of 0.5) and averaged over the nuclear area. Two types of experiments were performed: nuclei in different categories of deformation (based on their shapes and visual classification) were measured once, and single nuclei were measured repeatedly during time (once every hour for a maximum of 7 h).

### Numerical Modeling of Cell and Nuclear Deformations

A 2D computational model describing cellular deformation on microgrooves was developed using the commercial finite element software COMSOL Multiphysics 6.0. More detailed information on the modeling can be found in the Supporting Information. Briefly, the cell was described as a fluid‐filled elastic membrane, and cell deformation was computed based on a fluid‐structure interaction (FSI) formulation.^[^ ^44^, ^45^ ^]^ More specifically, a 0.2 µm‐thick cell membrane was considered a hyperelastic material described by an Odgen formulation.^[^ ^46^ ^]^ The cytoplasm was modeled as a Newtonian incompressible fluid^[^ ^47^ ^]^ with rheological characteristics similar to those of water. The microgroove PDMS substrate was treated as a non‐deformable structure since PDMS is considerably more rigid than cells. The movement of the cytoplasm was governed by the Navier–Stokes equations that describe mass and linear momentum conservation. The displacement field of the cell membrane was evaluated by solving the equation of motion.

The initial cell shape was considered to be an ellipse and two different sizes were considered: major axis of 15 µm, minor axis of 6.5 µm and major axis of 18 µm, minor axis of 8 µm. Cellular deformation and spreading over the substrate were driven by both membrane adhesion to the microgrooves and cell protrusion. Membrane‐adhesion to the microgroove surface was modeled based on a distance criterion *d* = 0.5 µm below which the cell membrane becomes attached to the substrate. Cell protrusion was modeled as a constant negative pressure *P_p_* = 10 Pa^[^ ^48^ ^]^ applied at the lower boundary of the cell. In addition to adhesion, membrane decohesion was also included to allow the cell to move into the microgrooves if the protrusion force was sufficiently large.

Similar to cells, isolated nuclei were modeled as fluid‐filled elastic membranes with an FSI formulation. The nuclei were assumed to initially have an elliptical shape with a major axis of 6 µm and a minor axis of 4 µm. Nuclear deformation on the microgroove substrate was assumed to be driven by gravity and nuclear envelope adhesion to the microgroove surface. As in the case of the cell, nuclear envelope decohesion was included, but it was characterized by a much lower maximum tensile stress and shear stress than cell decohesion to model weaker non‐specific adhesion. No protrusion pressure was considered in that case.

### Data Analysis—Analysis and Classifications of Nuclear Shapes

Detection and morphometric analysis of nuclei were performed using a custom‐made Matlab code. Briefly, DAPI staining images were used and binarized for subsequent detection of the nuclei. Different morphological parameters were extracted: nuclear (projected) area, nuclear aspect ratio (ratio of major to minor axis length), and measures of tortuosity such as solidity (ratio of area to convex area). From this morphometric analysis, caged nuclei were classified using the following criteria: minor axis < 7 µm and absolute orientation angle < 6° (0° being the orientation of the grooves). Nuclear volume was analyzed independently using the IMARIS software.

### Data Analysis—Analysis of Cytoskeletal Organization around Caged Nuclei

Caged nuclei were rotated vertically and cropped from confocal stacks. For each frame in the z direction, a plot profile of the fluorescence intensity across the nucleus minor axis (x‐axis) of the different cytoskeletal elements was generated and averaged over the entire nuclear length (y‐axis). The plot profile as a function of depth was then calculated by averaging all grey values for each z and normalizing by the maximum intensity.

### Data Analysis—Analysis of Nuclear Dynamics

To analyze nuclear dynamics, a fully automated pipeline was developed in Python. Hoechst images from the recordings were first pre‐processed using total variation denoising with a weight w = 0.01. Nuclei were then segmented from the preprocessed images by using a custom‐trained Cellpose v2 cytoplasm model.^[^ ^49^ ^]^ The morphological properties of each nucleus were extracted from the instance segmentations using the sci‐kit‐image library. Caged nuclei were classified based on the following criteria: minor axis of the fitted ellipse < 9.75 µm, absolute orientation angle < 6°, extent ≥ 0.75 (nucleus area divided by bounding box area), ratio between the major and minor axis length of the fitted ellipse > 2.0. Subsequently, nuclear tracking was performed using Trackmate and the PyImageJ library. Caging classifications were then integrated with the tracking data. In this analysis, the caging frequency was defined as the number of caging phases for HUVECs or transitions to an adjacent groove for myoblasts, divided by the total time of tracking.

### Data Representation and Statistical Analysis

In all boxplots, the central bar represents the median, the bottom and top edges of the box indicate the 25th and 75th percentiles, respectively, and the whiskers denote the range of minimum to maximum values, excluding outliers. All analyses were based on at least 3 independent experiments. Statistical analyses were performed using the GraphPad Prism software. The student *t*‐test and the Mann–Whitney *t*‐test were used to compare null hypotheses between two groups for normally and non‐normally distributed data, respectively. Multiple groups with a normal distribution were compared by a one‐way ANOVA followed by Tukey's or Fisher's post hoc test. The number of data points for each experiment, the specific statistical tests, and the significance levels are noted in the corresponding figure legends. On graphs, significance levels are represented with asterisks. In the case of multiple conditions, the colors of the asterisks match the condition to which the statistical comparison was made, progressing from left to right.

## Conflict of Interest

The authors declare no conflict of interest.

## Supporting information



Supporting Information

Supplemental Movie 1

Supplemental Movie 2

Supplemental Movie 3

Supplemental Movie 4

## Data Availability

The data that support the findings of this study are available from the corresponding author upon reasonable request.
