# Supplementary material for: Micro‐Scale Topography Triggers Dynamic 3D Nuclear Deformations
Source: Adv Sci (Weinh). 2025 Jan 28;12(11):2410052. doi: 10.1002/advs.202410052 (PMC11923911; doi:10.1002/advs.202410052)
Supplement: Supplementary file 1 — Supporting Information [file ADVS-12-2410052-s003.docx]

Supporting Information

**Micro-scale topography triggers dynamic 3D nuclear deformations**

*Claire Leclech*, Giulia Cardillo, Bettina Roellinger, Xingjian Zhang, Joni Frederick, Kamel Mamchaoui, Catherine Coirault and Abdul I. Barakat**

**Supplementary Figures**

**
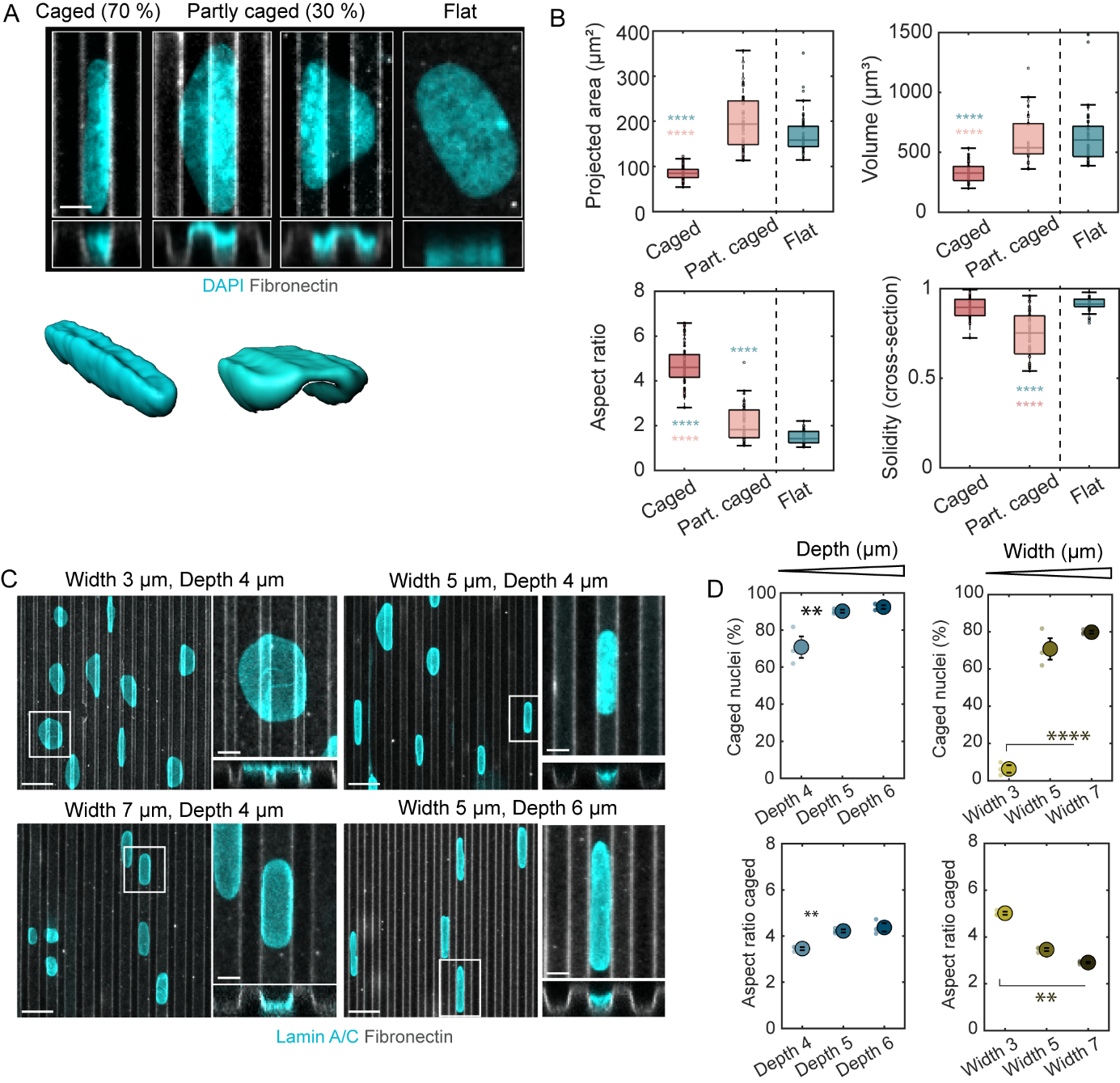
**

**Figure S1. Characterization of nuclear deformations on microgrooves in myoblasts.**

**(A)** Z-projection images, cross-sections and 3D reconstructions of the different classes of nuclei observed on microgrooves with DAPI. Scale bar 5 µm. **(B)** Morphological characterization of the different classes of nuclei observed on microgrooves and on control flat surfaces. Projected area and aspect ratio were quantified on z-projections images, solidity (tortuosity) on the cross-section images, and volume on 3D reconstructions. n=28-66 cells/category from 3 independent experiments. **(C)** Nuclei (stained for lamin A/C, cyan) on microgrooves (grey) of different dimensions. Scale bars 10 µm, 5 µm (zoom-ins). **(D)** Quantification of the percentage of caged nuclei and aspect ratio of caged nuclei for different groove depths (width = spacing = 5 µm) or groove widths (spacing = 5 µm, depth = 4 µm). Dots represent individual experiments and error bars represent standard error of the mean (SEM). n=3 independent experiments. For all plots: one-way ANOVA, Fisher's post-test (** p < 0.01; **** p < 0.0001).


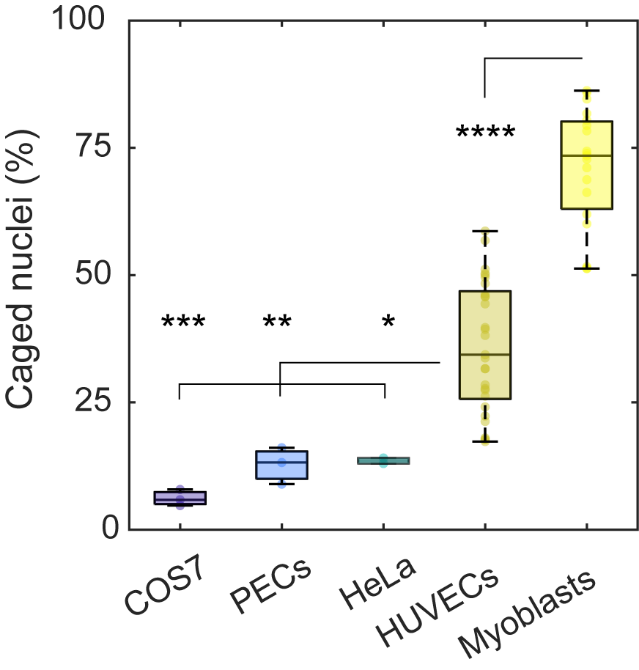


**Figure S2. Incidence of nuclear caging for different cell types.**

Percentages of caged nuclei for COS7 (n=3 independent experiments), PECs (n=3 independent experiments), HeLa cells (n=2 independent experiments), HUVECs (n=29 independent experiments), and myoblasts (n=19 independent experiments). One-way ANOVA, Fisher's post-test (* p = 0.011; ** p = 0.018; ** p = 0.0001; **** p < 0.0001).


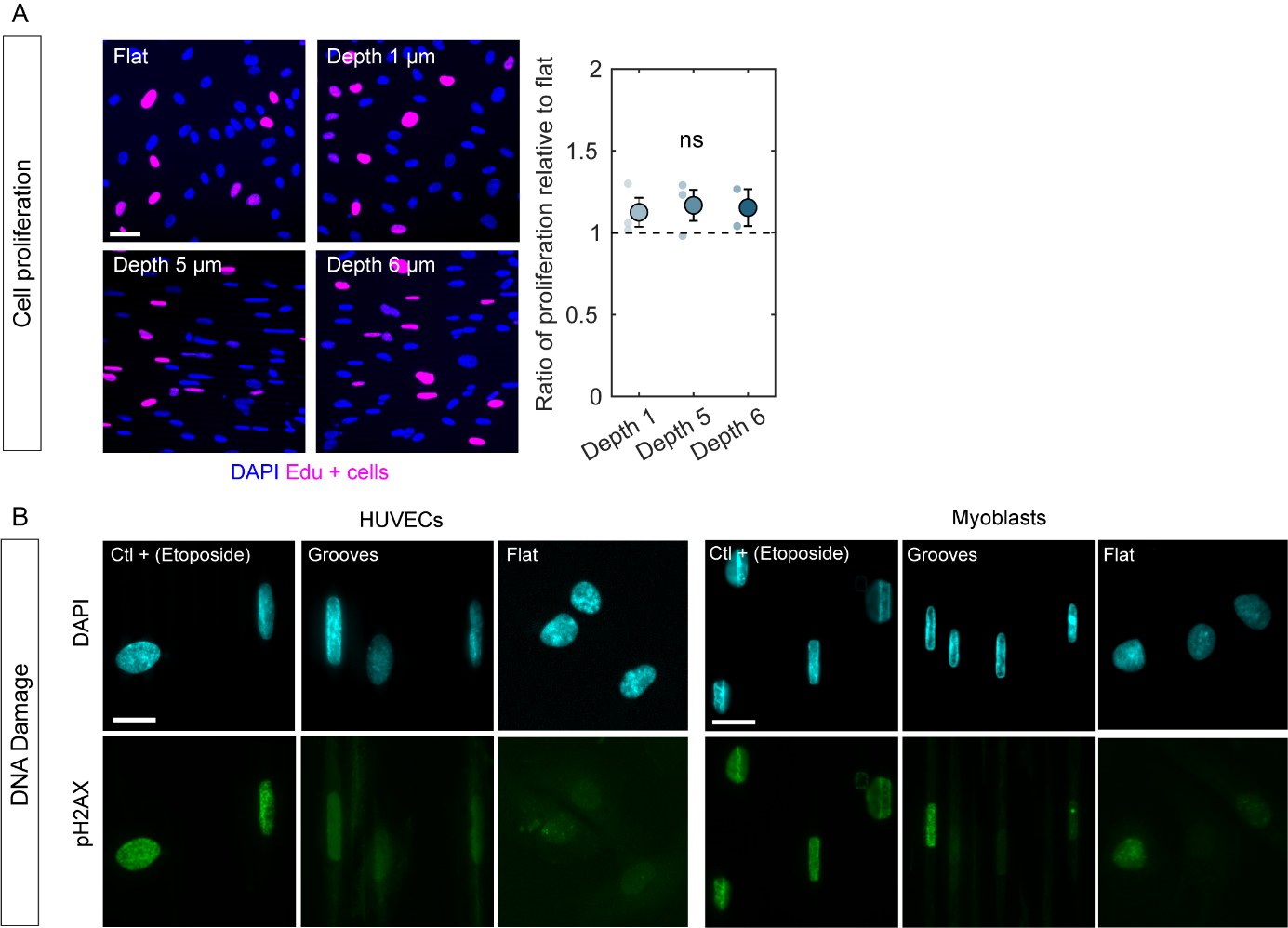


**Figure S3. Functional consequences of nuclear deformations on microgrooves.**

**(A)** EdU assay was used to assess cell proliferation for different groove depths compared to flat PDMS surfaces. Quantification shows the ratio of the percentage of EdU-positive cells on microgrooves to those on flat substrates. **(B)** Immunostaining against phospho-H2AX was used to assess DNA damage in HUVECs or myoblasts cultured on microgrooves or on flat PDMS surfaces. For a positive control, cells were incubated with etoposide (50 mM) for 3 h to induce chemical DNA damage. Scale bars 20 µm.


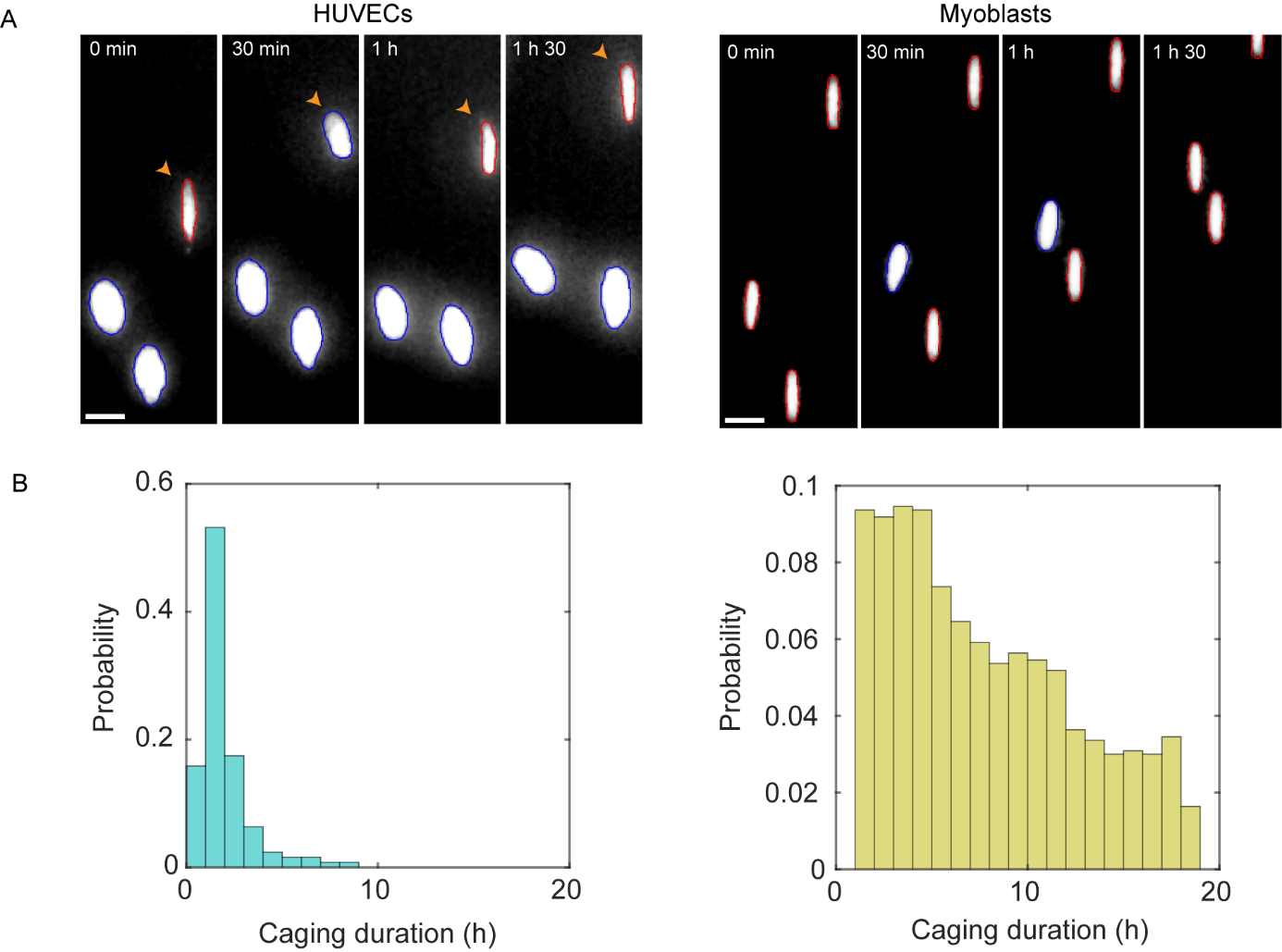


**Figure S4. Automatic detection of caged phases in recordings.**

**(A)** Output of the automatic detection of caged (red) or uncaged (blue) phases in recording of HUVEC or myoblast nuclei. Scale bar, 20 µm. **(B)** Distributions of caging duration for HUVECs (left; n=126 cells) and myoblasts (right; n=1099 cells).


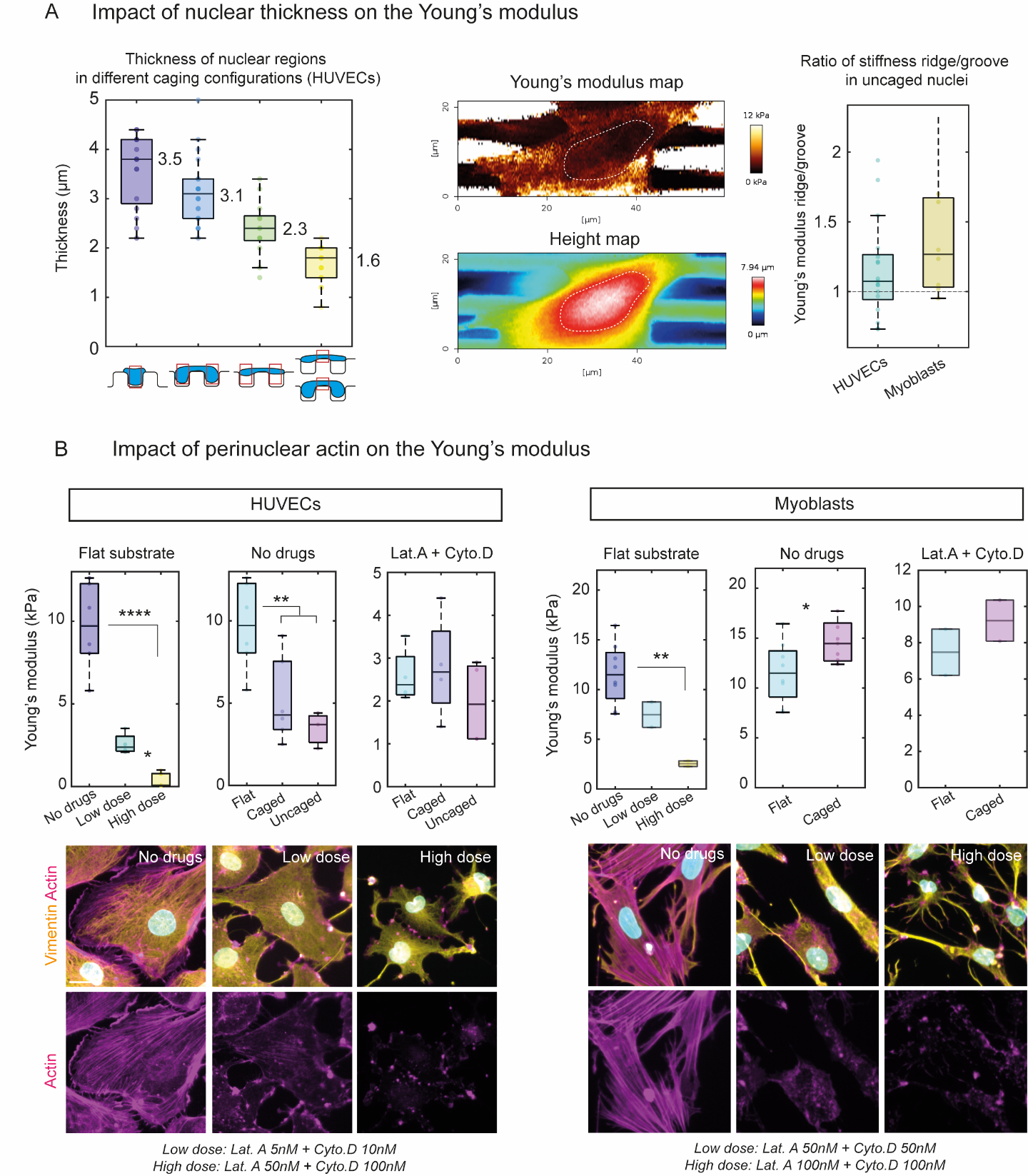


**Figure S5. Influence of nuclear thickness and perinuclear actin on AFM measurements.**

**(A)** Left: nuclear height in different deformation states from confocal images (HUVECs, 13 to 22 nuclei/category from one experiment). Middle: AFM output for Young’s modulus map and corresponding height map of a HUVEC lying both on ridges and grooves. Position of the nucleus is shown with the white dotted contour. Right: in uncaged nuclei, ratio of the Young’s modulus measured on the ridge to that on the groove (HUVECs: 17 nuclei from 3 independent experiments, myoblasts: 8 nuclei from 2 independent experiments). **(B)** AFM measurements on cells treated with latrunculin A and cytochalasin D. Left panels show the impact of different drug doses on the perinuclear Young’s moduli on flat substrates. The other panels show the perinuclear Young’s moduli for cells on flat substrates and for caged (or uncaged) nuclear configurations without or with drugs (low dose). n=2 to 5 independent experiments with at least 11 cells/category. One-way ANOVA, Fisher's post-test (3 groups) or Student t test (2 groups) (* p = 0.03; ** p = 0.018; ** p < 0.008; **** p < 0.0001). Immunostaining images show the effect of the different drug concentrations on the cells on flat surfaces. Scale bar 20 µm.


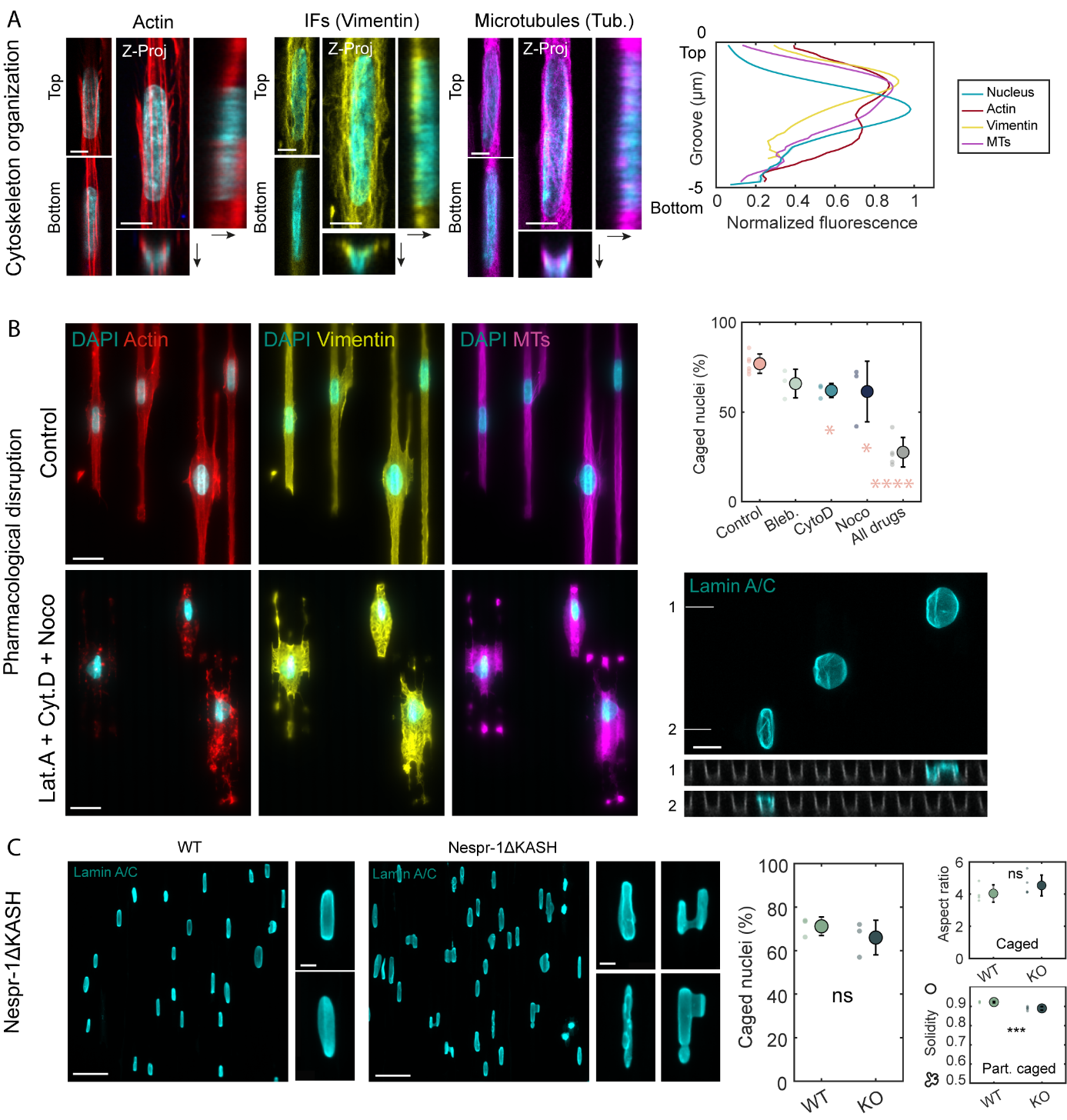


**Figure S6. Organization and role of the cytoskeleton in nuclear deformations on microgrooves in myoblasts.**

**(A) Left:** Z-projections and cross-sections showing the organization of actin (red), intermediate filaments (vimentin, yellow), and microtubules (magenta) around caged nuclei. Scale bars 5 µm. **Right:** Quantification of the normalized fluorescence intensity for the three cytoskeletal networks and the nucleus as a function of depth (from top to bottom of the grooves) for caged nuclei. **(B)** **Left:** Immunostaining for actin (red), intermediate filaments (vimentin, yellow), and microtubules (magenta) in control cells or cells treated with latrunculin A (Lat.A) + cytochalasin D (Cyto.D) + nocodazole (Noco) on microgrooves (5x5x5 µm, vertical). Scale bar 20 µm. **Right:**  Quantification of the percentage of caged nuclei for the different pharmacological treatments: control (DMSO), blebbistatin (Bleb.), cytochalasin D (Cyto.D), nocodazole (Noco), or latrunculin A + cytochalasin D + nocodazole (All drugs). Dots represent individual experiments and error bars represent standard deviations. n=3 to 7 independent experiments. One-way ANOVA, Fisher's post-test (* p < 0.1; **** p < 0.0001). Z-projection and cross-sections of nuclei stained for lamin A/C and treated with latrunculin A + cytochalasin D + nocodazole. Scale bar 10 µm. **(C)** WT or Nesprin1-mutated (Nespr-1ΔKASH) nuclei on microgrooves stained for lamin A/C. Scale bars 50 µm, 5 µm (insets). Quantification of the percentage of caged nuclei, aspect ratio of caged nuclei, and solidity of partly caged nuclei. Dots represent individual experiments and error bars represent standard deviations. n=3 independent experiments. Student t test (* p < 0.1; ** p < 0.01; *** p < 0.001; **** p < 0.001).


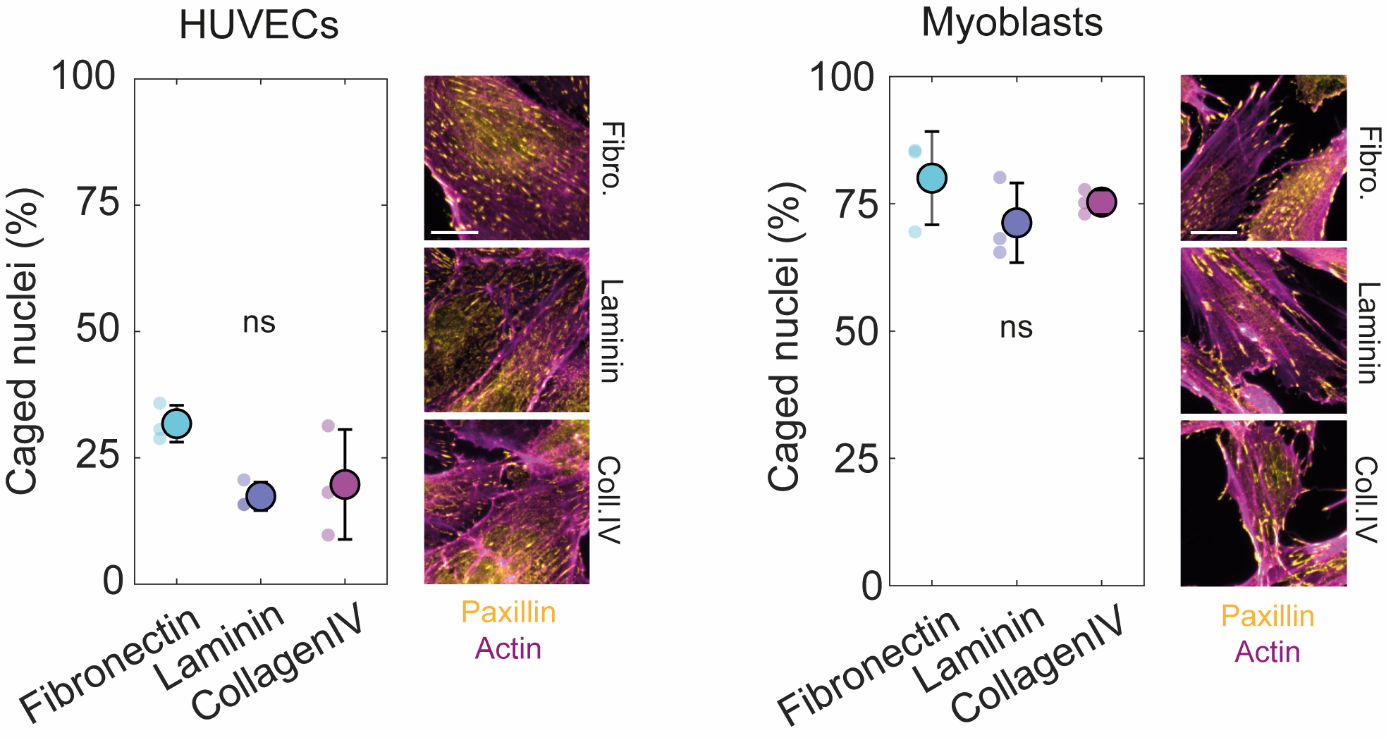


**Figure S7. Nuclear caging for different types of protein coatings.**

Percentage of caged nuclei on cells cultured overnight on microgrooves (5x5x5 µm) coated with fibronectin, laminin, or collagen IV. n=3 independent experiments. One-way ANOVA, Fisher's post-test (ns, p > 0.1). Images show immunostaining of focal adhesions (paxillin, yellow) and actin (phalloidin, magenta) for the different coatings on flat surfaces. Scale bar 20 µm.

**
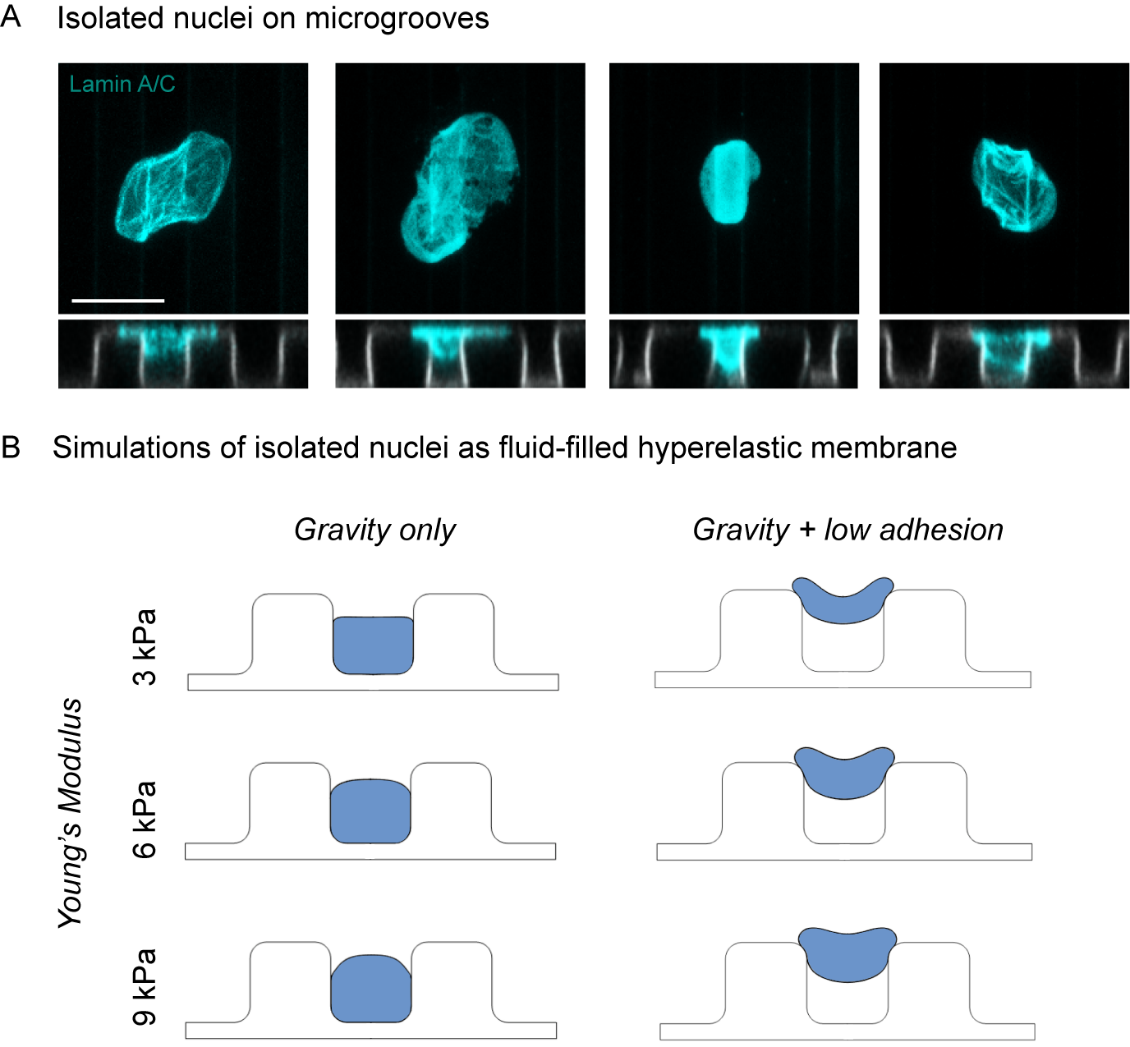
**

**Figure S8. Behavior of isolated nuclei on microgrooves.**

**(A)** Z-projections and cross-sections of 4 different isolated nuclei deposited on microgrooves and stained for lamin A/C. Scale bar 10 µm. **(B)** Numerical simulations of nuclei deformations on microgrooves for different Young’s Moduli, considering the effect of gravity only or gravity and a low adhesion force to the substrate.


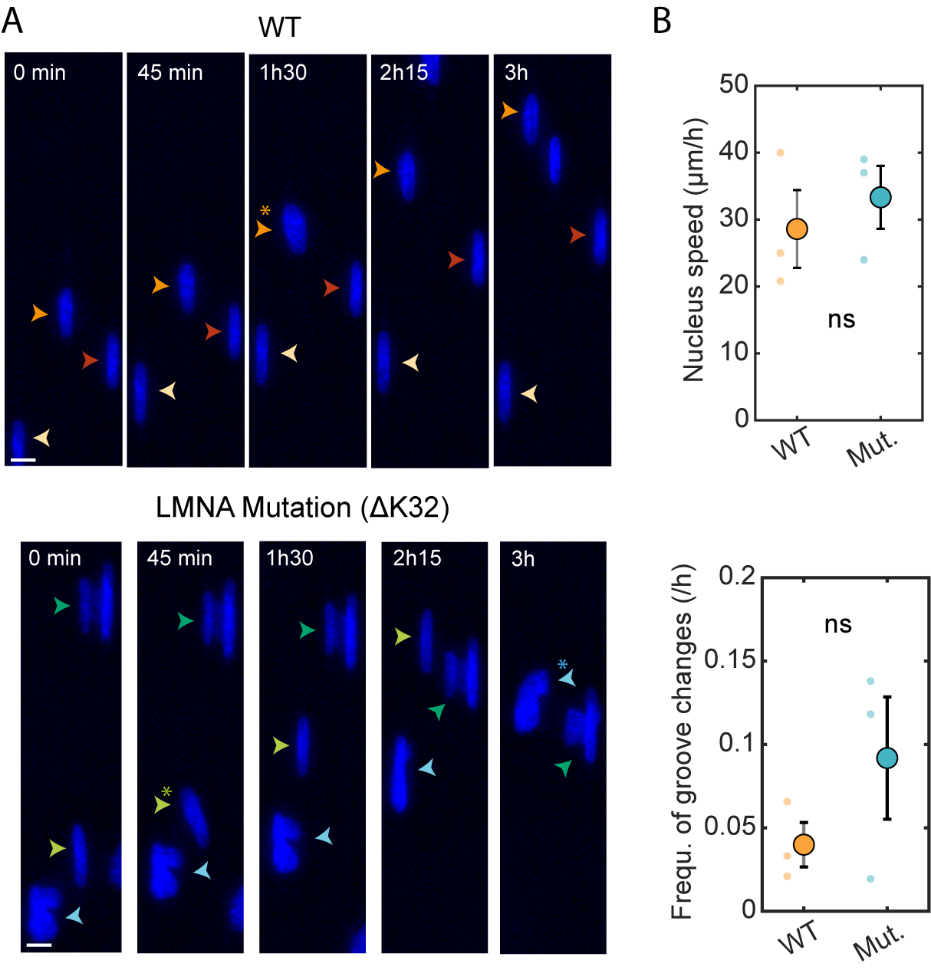


**Figure S9. Dynamics of LMNA mutated myoblasts on microgrooves.**

**(A)** Images extracted from time-lapse recordings of WT or mutated (LMNA mutation ΔK32) myoblast nuclei (stained with Hoechst, blue) on microgrooves (5x5x5 µm, vertical). Arrowheads follow the path of a single nucleus, and stars indicate uncaging phases. Scale bar 10 µm. **(B)** Quantification of the mean nucleus speed and frequency of groove changes. Dots represent individual experiments and error bars represent standard error of the mean (SEM). n=3 independent experiments. Student t test.

**Movie S1.**

Time lapse recording of a HUVEC with nuclei stained with Hoechst on microgrooves (5x5x5 µm, vertical). Time interval 15 min, scale bar 20 µm.

**Movie S2.**

Time lapse recording of a myoblast with nuclei stained with Hoechst on microgrooves (5x5x5 µm, vertical). Time interval 15 min, scale bar 20 µm.

**Movie S3.**

Time lapse recording of a HUVEC with nuclei stained with Hoechst and cell membrane stained with CellMask, on microgrooves (5x5x5 µm, vertical), showing correlated cell and nuclear behavior. Time interval 5 min, scale bar 20 µm.

**Movie S4.**

Time lapse recording of a HUVEC with nuclei stained with Hoechst and cell membrane stained with CellMask, on microgrooves (5x5x5 µm, vertical), showing decorrelated cell and nuclear behavior. Time interval 5 min, scale bar 20 µm.

**Supplementary Text: Computational model methods**

A 2D computational model describing cellular deformation on microgrooves was developed using the commercial finite element software COMSOL Multiphysics 6.0. In the model, the cell is described as a fluid-filled elastic membrane, and cell deformation is computed based on a fluid-structure interaction (FSI) formulation ^[1,2]^. More specifically, in line with experimental evidence, a 0.05 µm-thick cell membrane is considered as a hyperelastic material described by an Odgen formulation ^[3]^. The cytoplasm is modeled as a Newtonian incompressible fluid ^[4]^ with rheological characteristics similar to those of water. The microgroove PDMS substrate is treated as a non-deformable structure since PDMS is considerably more rigid than cells.

In the model, the movement of the cytoplasm is governed by the Navier-Stokes equations that describe mass and linear momentum conservation (Eqs. 1 and 2):

$\nabla\cdot\vec{v}=0$ (1)

$\rho\frac{\partial\vec{v}}{\partial t}=-\nabla p+\rho\vec{g}+\mu\nabla^{2}\vec{v,}$ (2)

where ρ is the cytoplasm density, $\vec{v}$ and *p* are the fluid velocity vector and pressure, respectively, and µ is the cytoplasm dynamic viscosity.

The displacement field of the cell membrane is evaluated by solving the equation of motion:

$\rho_{m}\frac{\partial^{2}\vec{u}}{\partial t^{2}}=\nabla\cdot{(FS)}^{T}+\vec{F_{v}}$*,* (3)

where *F=I+*$\nabla\vec{u}$, with I denoting the unit diagonal matrix, $\vec{u}$ the displacement of the membrane,$\rho_{m}$ the density of the membrane, S the second Piola–Kirchhoff stress tensor, and $\vec{F_{v}},$ any externally applied force on the membrane.

At the interface between the cytoplasm and the membrane, i.e. at the fluid-solid interface, the continuity of stresses is imposed as follows:

$\vec{F_{n}}=-\vec{n}\cdot(-pI+\mu\left( \nabla\vec{v}+{(\nabla\vec{v})}^{T} \right)$ (4)

$\vec{v}=\frac{\partial\vec{u}}{\partial t}.$ (5)

Eq. (4) ensures that the normal force $\vec{F_{n}}$ acting on the membrane at the fluid-solid interface is

equal to the force acting on the cytoplasm. Eq. (5) ensures that at the interface, the cytoplasmic fluid velocity and the velocity of the membrane are identical. Due to the low velocities that characterize the problem, inertial terms were neglected in both the Navier-Stokes equations and the equation of motion.

In the model, the initial cell shape is considered to be an ellipse and two different sizes were considered, one with a major axis of 12.5 µm and a minor axis of 6.5 µm and the other with a major axis of 18 µm and a minor axis of 8 µm. In both cases, the ellipse was positioned in contact with the microgroove substrate. Cellular deformation and spreading over the substrate are driven by both membrane adhesion to the microgrooves and by cell protrusion. Membrane- adhesion to the microgroove surface is modeled based on a distance criterion. Thus, the cell membrane becomes attached to the substrate if the distance between the membrane and the substrate is smaller than a critical value *d*. Cell protrusion was modeled as a constant negative pressure *P_p_* ^[5]^ applied at the lower boundary of the cell as shown in Fig. 1. In addition to adhesion, membrane decohesion was also included in order to allow the cell to move into the microgrooves if the protrusion force is sufficiently large. The criterion for decohesion is defined based on the maximum tensile stress *σ_c_* and shear stress*τ_c_* that the adherent membrane can withstand before detachment. A stationary study was implemented, and both the protrusion pressure *Pp* and the critical distance *d* at which membrane- adhesion occurs were ramped very slowly (0.005 steps) to improve convergence.


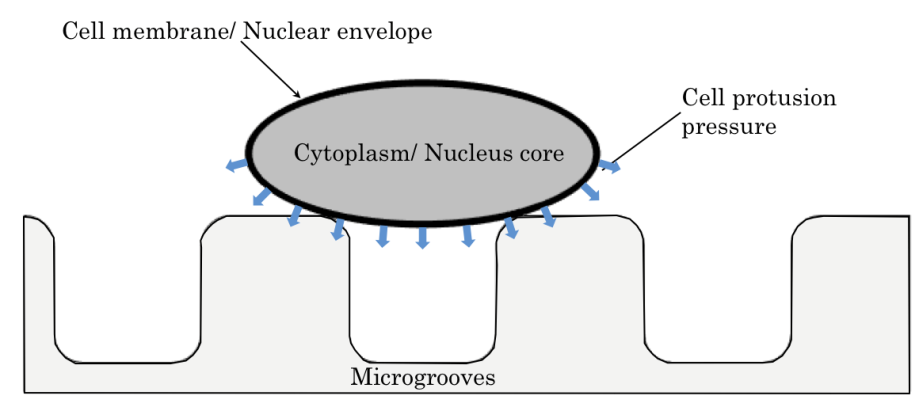


Figure 1 : Modeling scheme

The microgroove domain was spatially discretized in 1,450 fixed triangular mesh elements. For the cell, a very fine deformable mesh consisting of 19,000 to 42,000 triangular elements was considered for both the fluid and the membrane.

Similar to cells, isolated nuclei were modeled as fluid-filled elastic membranes with an FSI formulation. The nuclei were assumed to initially have an elliptical shape with a major axis of 6 µm and a minor axis of 4 µm. The thickness of the nuclear envelope was set to 0.04 µm ^[6]^. Nuclear deformation on the microgroove substrate is assumed to be driven by both gravity and nuclear envelope adhesion to the microgroove surface. The latter was modeled based on a distance criterion, considering the same critical distance *d* imposed for cell modeling. As in the case of the cell, nuclear envelope decohesion was included, but it was characterized by a much lower maximum tensile stress *σ_n_* and shear stress *τ_n_* than cell decohesion in order to model weaker non-specific adhesion. No protrusion pressure was considered in the case of the nucleus. As in the case of the cell deformation model, a stationary study was implemented with both the gravity force and the critical distance *d* at which nuclear envelope- adhesion takes place ramped very slowly (0.001 steps) to improve convergence. In the nucleus deformation model, the microgroove domain was spatially discretized in 1,450 fixed triangular mesh elements. For the nucleus, a very fine deformable mesh consisting of 9,975 triangular elements was considered.

The displacement of the mesh was implemented using the arbitrary Lagrangian–Eulerian (ALE) description in modeling both cell and nuclear deformations. A segregated Newton nonlinear solver was employed to solve the equations. The computational times were typically on the order of 45 min on an Intel(R) Core(TM) i7-10810U machine, with clock frequency of 1.1 GHz. Mesh independence was verified for all simulations. The model parameter values are reported in Table 1.

Table 1 :Values of the model parameters

| Parameter | Value | Unit | Reference |
| --- | --- | --- | --- |
| Young’s modulus of the cell membrane | 3,500 | Pa | ^[7]^ |
| Poisson’s coefficient of the cell membrane | 0.5 | [-] | ^[8]^ |
| Alpha parameter in Odgen formulation for the cell membrane | 1.5 | [-] | ^[3]^ |
| Cell membrane density | 1,100 | kg/m^3^ | ^[9]^ |
| Cytoplasm density | 1,080 | kg/m^3^ | ^[9]^ |
| Cytoplasm viscosity | 0.001 | Pa∙s | ^[9]^ |
| Young’s modulus of the nuclear envelope | 5,250 | Pa | ^[10]^ |
| Poisson’s coefficient of the nuclear envelope | 1.5 | [-] | ^[9]^ |
| Nuclear density | 2,000 | kg/m^3^ | ^[9]^ |
| Nuclear viscosity | 0.01 | Pa∙s |  |
| Nuclear envelope density | 1,300 | kg/m^3^ | ^[9]^ |
| Critical value *d* | 0.5 | µm |  |
| Protrusion pressure *P_p_* | 10 | Pa | ^[5]^ |
| Maximum tensile stress for cell decohesion *σ_c_* | 10 | Pa |  |
| Maximum shear stress for cell decohesion *τ_c_* | 5 | Pa |  |
| Maximum tensile stress for nucleus decohesion *σ_n_* | 0.3 | Pa |  |
| Maximum shear stress for cell decohesion *τ_n_* | 0.15 | Pa |  |

References

[1] L. Santoro, L. Vaiani, A. Boccaccio, L. Lamberti, L. Lo Muzio, A. Ballini, S. Cantore, *Appl. Sci.* **2024**, *14*, 2596.

[2] S. M. McFaul, B. K. Lin, H. Ma, *Lab Chip* **2012**, *12*, 2369.

[3] J. Chen, J. Irianto, S. Inamdar, P. Pravincumar, D. A. Lee, D. L. Bader, M. M. Knight, *Biophys. J.* **2012**, *103*, 1188.

[4] R. Niwayama, K. Shinohara, A. Kimura, *Proc. Natl. Acad. Sci.* **2011**, *108*, 11900.

[5] T. E. Woolley, E. A. Gaffney, J. M. Oliver, R. E. Baker, S. L. Waters, A. Goriely, *Biomech. Model. Mechanobiol.* **2014**, *13*, 463.

[6] A. M. Chizhik, D. Ruhlandt, J. Pfaff, N. Karedla, A. I. Chizhik, I. Gregor, R. H. Kehlenbach, J. Enderlein, *ACS Nano* **2017**, *11*, 11839.

[7] H. Oberleithner, C. Riethmüller, T. Ludwig, V. Shahin, C. Stock, A. Schwab, M. Hausberg, K. Kusche, H. Schillers, *J. Cell Sci.* **2006**, *119*, 1926.

[8] N. Marín-Medina, D. A. Ramírez, S. Trier, C. Leidy, *Appl. Microbiol. Biotechnol.* **2016**, *100*, 10251.

[9] A. Geltmeier, B. Rinner, D. Bade, K. Meditz, R. Witt, U. Bicker, C. Bludszuweit-Philipp, P. Maier, *PLoS One* **2015**, *10*, e0134999.

[10] K. Wang, Y. Qin, Y. Chen, *Biochim. Biophys. Acta - Mol. Cell Res.* **2021**, *1868*, 118985.
